# Denervation during mandibular distraction osteogenesis results in impaired bone formation

**DOI:** 10.1038/s41598-023-27921-9

**Published:** 2023-02-06

**Authors:** Ruth Tevlin, Michelle Griffin, Kellen Chen, Michael Januszyk, Nick Guardino, Amanda Spielman, Shannon Walters, Garry Evan Gold, Charles K. F. Chan, Geoffrey C. Gurtner, Derrick C. Wan, Michael T. Longaker

**Affiliations:** 1 Division of Plastic and Reconstructive Surgery, Stanford Hospital and Clinics, Stanford, CA USA; 2 School of Postgraduate Studies, Royal College of Surgeons in Ireland, Dublin, Ireland; 3 Hagey Laboratory for Pediatric Regenerative Medicine, Department of Surgery, Stanford University School of Medicine, 257 Campus Drive, Stanford, CA 94305-5418 USA; 4 Department of Radiology, Stanford University School of Medicine, 1201 Welch Road P263, Stanford, CA 94305 USA; 5 Institute for Stem Cell Biology and Regenerative Medicine, Stanford University School of Medicine, Stanford, CA USA; 6 School of Medicine, Stanford University, Stanford, CA USA; 7 Department of Surgery, University of Arizona Health Sciences, Tucson, AZ USA

**Keywords:** Regeneration, Targeted bone remodelling, Bone

## Abstract

Mandibular distraction osteogenesis (DO) is mediated by skeletal stem cells (SSCs) in mice, which enact bone regeneration via neural crest re-activation. As peripheral nerves are essential to progenitor function during development and in response to injury, we questioned if denervation impairs mandibular DO. C57Bl6 mice were divided into two groups: DO with a segmental defect in the inferior alveolar nerve (IAN) at the time of mandibular osteotomy (“DO Den”) and DO with IAN intact (“DO Inn”). DO Den demonstrated significantly reduced histological and radiological osteogenesis relative to DO Inn. Denervation preceding DO results in reduced SSC amplification and osteogenic potential in mice. Single cell RNA sequencing analysis revealed that there was a predominance of innervated SSCs in clusters dominated by pathways related to bone formation. A rare human patient specimen was also analyzed and suggested that histological, radiological, and transcriptional alterations seen in mouse DO may be conserved in the setting of denervated human mandible distraction. Fibromodulin (FMOD) transcriptional and protein expression were reduced in denervated relative to innervated mouse and human mandible regenerate. Finally, when exogenous FMOD was added to DO-Den and DO-Inn SSCs undergoing in vitro osteogenic differentiation, the osteogenic potential of DO-Den SSCs was increased in comparison to control untreated DO-Den SSCs, modeling the superior osteogenic potential of DO-Inn SSCs.

## Introduction

Distraction osteogenesis (DO) is a regenerative surgical technique whereby new bone is formed between surgically osteotomized bone segments undergoing gradual separation by incremental traction, enabling greater corrections in bone position and complementary soft tissue expansion than are possible in a single surgery. Since the first reported clinical application of DO in the craniofacial skeleton in 1989^1^, there has been a substantial expansion of the indications for DO in craniofacial surgery. For example, DO is now used to promote bone in a variety of conditions with congenital skeletal deficiency (e.g. Treacher Collins Syndrome, Pierre Robin Sequence, and hemifacial microsomia), resulting in reduced rates of airway obstruction and tracheostomy, and improved health-related quality of life^2–5^.

The peripheral nervous system has been intimately linked to bone metabolism, osteogenic differentiation of stem/progenitor cells and bone remodeling^6^. In amphibians, innervated limbs regenerate after proximal amputation, whereas denervation precludes regeneration leading to stump formation^7^. Further experimental studies have shown that peripheral nerve fibers influence fracture repair in rats^8^. Sympathetic and sensory nerve fibers innervate periosteum, trabecular bone and fracture callus, and have also been shown to impact vascularization, embryonic limb osteogenesis and bone regeneration^9–11^. Focusing on the mandible, our laboratory has previously demonstrated that uni-cortical mandibular bone fracture repair is deficient in the setting of denervation and can be rescued by Schwann cell transplantation^12^. Implementing an established mouse model of mandible DO^13^, we sought to determine whether the regenerative capacity of DO is compromised in the setting of denervation. The mandible and inferior alveolar nerve (IAN) have an intimate relationship, as the nerve runs directly through the bone in the mandibular canal, thus, offering a unique window to investigate the influence of innervation on DO.

In 2015, Chan and colleagues characterized the mouse skeletal stem cell (mSSC), a single cell capable of giving rise to bone, cartilage and bone marrow stroma, and which has an established downstream differentiation to osteo-, chondro-, and stromal progenitors^14^. Prior work by Ransom et al. demonstrated that newly formed bone following DO is clonally derived from stem cells that reside in the skeleton after reverting to a developmental neural crest-like state^13^. These findings from our group, together with our prior isolation of mSSCs from long bone^14,15,16^, long bone fractures^16–18^, and mandibles^12,13^, prompted our examination of the effect of denervation on SSC expansion and osteogenic potential in the setting of mandible DO. Harnessing the underlying biology of nerve dependent DO holds promise for clinical innovation in the management of traumatic and congenital craniofacial abnormalities. DO is a lengthy and cumbersome process for patients and their families, and can be complicated by post-operative skeletal relapse, whereby the final length of bone diminishes after treatment. If we can better understand the mechanisms driving DO, we can better treat children born with congenital skeletal anomalies and adults with acquired skeletal deficiency and improve health-related quality of life.

Here we develop a robust model of IAN denervation in the setting of mandible distraction. We use this model to interrogate the effect of denervation on histological and radiological bone regeneration. By prospective isolation of mSSCs, we demonstrate a reduced expansion of mSSCs together with downstream progenitor cells following DO. Transcriptional profiling by single cell RNA sequencing (scRNA seq) demonstrated that there was a predominance of innervated SSCs in clusters dominated by pathways related to bone formation. Intriguingly, a rare human patient specimen was also analyzed, and resultant data suggested that histological, radiological and transcriptional alterations seen in mouse DO may be conserved in the setting of denervated human mandible distraction three months following distraction. Fibromodulin (FMOD) transcriptional expression was reduced in denervated relative to innervated mouse mandible regenerate. Furthermore, relatively lower FMOD expression was detected using immunohistochemistry in both human and mouse denervated versus innervated bone regenerate. When exogenous FMOD was added to mouse DO-Den and DO-Inn SSCs undergoing in vitro osteogenic differentiation, the osteogenic potential of DO-Den SSCs was increased in comparison to control untreated DO-Den SSCs, modeling the superior osteogenic potential of DO-Inn SSCs. Together these data suggest that peripheral nerves maintain a salient role in DO by promoting non-neuronal (bone) regeneration and that this may be mediated by FMOD in both mice and humans.

## Results

### Development of a mouse model of mandibular denervation in the setting of DO

**Figure 1:**
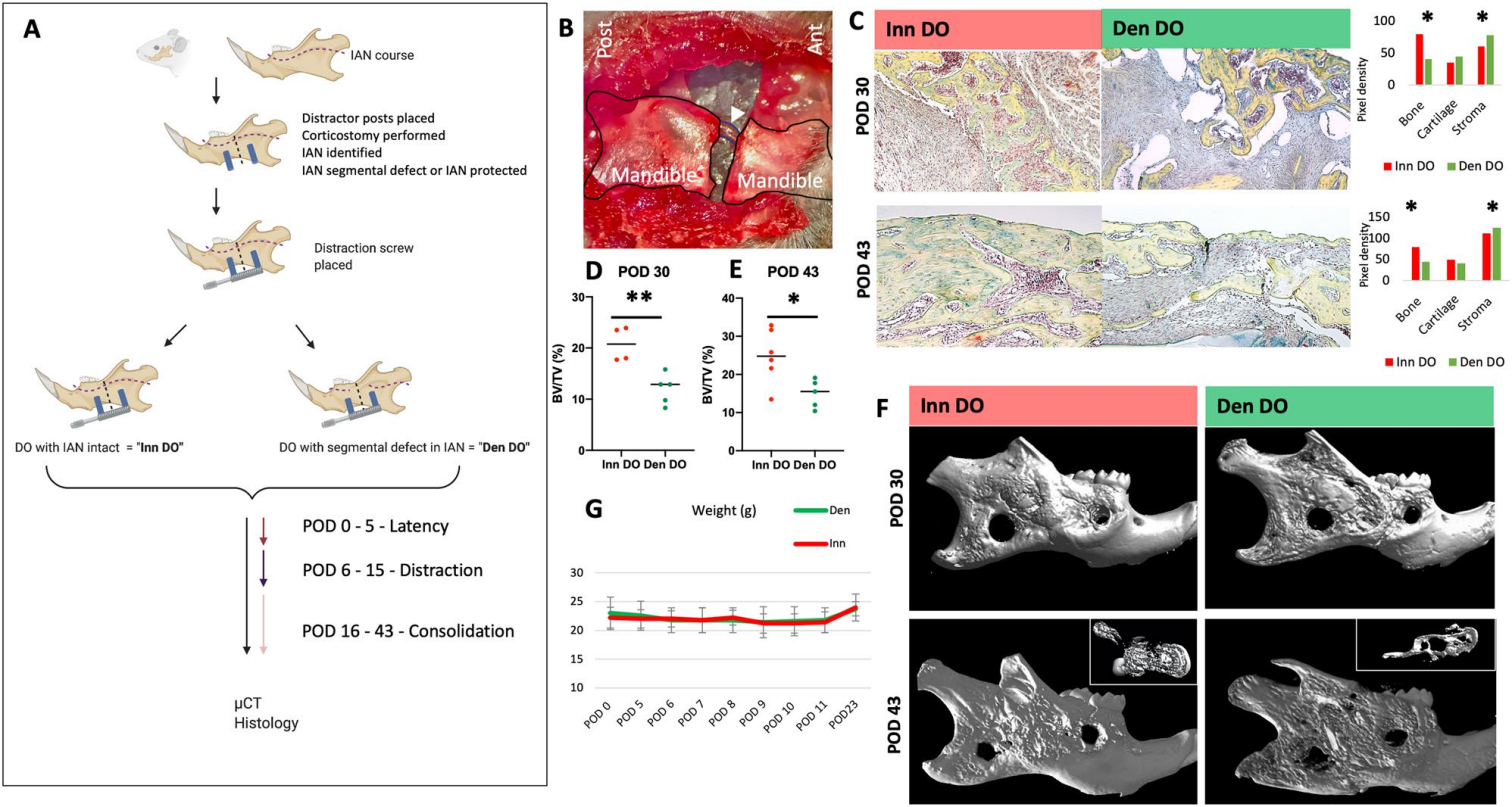
Denervation of IAN during DO leads to impaired histological and radiological osteogenesis. (**A**) Experimental schematic: C57Bl6 mice underwent distractor placement with or without a segmental resection (“segmental defect”) of the IAN. The mice then underwent 5 days of latency (no distraction), following by 10 days of distraction (POD 5–15), followed by consolidation until POD 43. For histological analysis of osteogenesis, mice were euthanized at specific timepoints and their mandibles underwent histological staining with Movat’s pentachrome. For radiological analysis of osteogenesis, mandibles underwent ex vivo CT scanning, followed by 3 dimensional μCT reconstruction and analysis of the bone regenerate callus mineralized volume fraction (bone volume / total tissue volume, or BV/TV). Created with BioRender.com. (**B**) Microsurgical image of mandible post osteotomy with intact IAN shown. Posterior mandible on left, anterior mandible on right. (Black = background plastic material to ensure improved visualization of IAN for photograph). (**C**) Movat’s pentachrome staining of coronal section of bone regenerate Inn DO (left) vs Den DO (middle) mandibles at the following time-points: POD 30 (top panels) and POD 43 (bottom panels). Orientation left—posterior, right—anterior, 10X. Here, yellow denotes bone formation, blue denotes cartilage formation, purple denotes fibrous stroma formation. (right): Graph demonstrating composition of regenerate (bone, cartilage or fibrous stroma) top = POD 30, bottom = POD 43. Here * denotes p < 0.05. (**D**) Graph demonstrating analysis of POD 30 bone regenerate callus mineralized volume fraction (bone volume/total tissue volume, or BV/TV) of Inn DO (red circles) versus Den DO (green circles) as determined by μCT. (**E**) Graph demonstrating analysis of POD 43 bone regenerate callus mineralized volume fraction (bone volume/total tissue volume, or BV/TV) of Inn DO (red circles) versus Den DO (green circles) as determined by μCT. (**F**) Representative 3-dimensional (3D) μCT reconstructions of Inn DO (left) vs Den DO (right) mandibles at the following time-points: POD 30 (top panels) and POD 43 (bottom panels). Inset demonstrates axial view of the mandible. (**G**) Graph demonstrating no significant difference in body weight (grams) of Den DO (green) and Inn DO (red) mice at multiple timepoints post-operatively.

Building on prior work which demonstrated that unicortical mandibular bone healing was impaired in the setting of denervation of the IAN^12^, we sought to determine whether the regenerative capacity of DO is compromised in the setting of denervation of the IAN. Thus, to interrogate the effect of denervation on mandibular DO, we devised a mouse model of segmental IAN injury at the time of mandibular osteotomy and distractor hardware placement (Fig. 1A). Here, using an operative microscope, a segmental (4 mm) defect of the IAN was performed following mandibular osteotomy in the denervated cohort (hereafter referred to as “Den DO”) of *C57Bl6* mice. In the control innervated *C57Bl6* group (hereafter referred to as “Inn DO”), the IAN was protected along its course (Fig. 1B). The mice then underwent 5 days of latency, followed by distraction on post-operative day (POD) 5 to 15, and consolidation from POD16 to POD 43, as previously reported^13^. The denervation procedure was then validated using *Thy1-YFP* mice who underwent Inn DO versus Den DO and were then imaged stereomicroscopically at POD 43. Confocal microscopy confirmed segmental injury of the IAN with significantly decreased *Thy1* expression in the IAN distribution following segmental IAN injury at the time of distractor placement in the Den DO relative to Inn DO cohort (Supplementary Fig. 1A).

### Segmental injury of the IAN prior to mandibular distraction results in reduced osteogenesis in mice

Following validation of the denervation injury, we next tested the effect of denervation on histological and radiological bone formation (Fig. 1C–F). Den DO and Inn DO mandibles were harvested at POD 30 and POD 43 and analyzed using micro-computed tomography (μCT) and Movat’s pentachrome histological staining. Movat’s pentachrome analysis revealed reduced bone formation and increased fibrous stroma at POD 30 and POD 43 in the Den DO relative to Inn DO cohorts (Fig. 1C). The bone regenerate callus mineralized volume fraction (bone volume/total tissue volume, or BV/TV) was also significantly reduced in Den DO relative to Inn DO when 3-dimensional μCT reconstructions were analyzed (POD 30 **p < 0.01; POD 43 *p < 0.05) (Fig. 1D–F, Supplementary Fig. 1B). Given the concern that a denervation injury could theoretically affect food intake leading to impaired osteogenesis, weights of Inn DO and Den DO mice were serially recorded with no significant difference seen (Fig. 1G). Furthermore, we then followed mice to POD 90 to determine whether the Den DO mandible osteogenesis was delayed and could theoretically “catch up” to that of the Inn DO bone over a prolonged time period. However, we again saw that bone regenerate callus mineralized volume fraction (bone volume/total tissue volume, or BV/TV) was also significantly reduced in Den DO relative to Inn DO when 3-dimensional μCT reconstructions were analyzed (POD 90 *p < 0.05) (Supplementary Fig. 1C,D).

### Mandible skeletal stem cell kinetics and osteogenic potential following DO are nerve dependent

**Figure 2:**
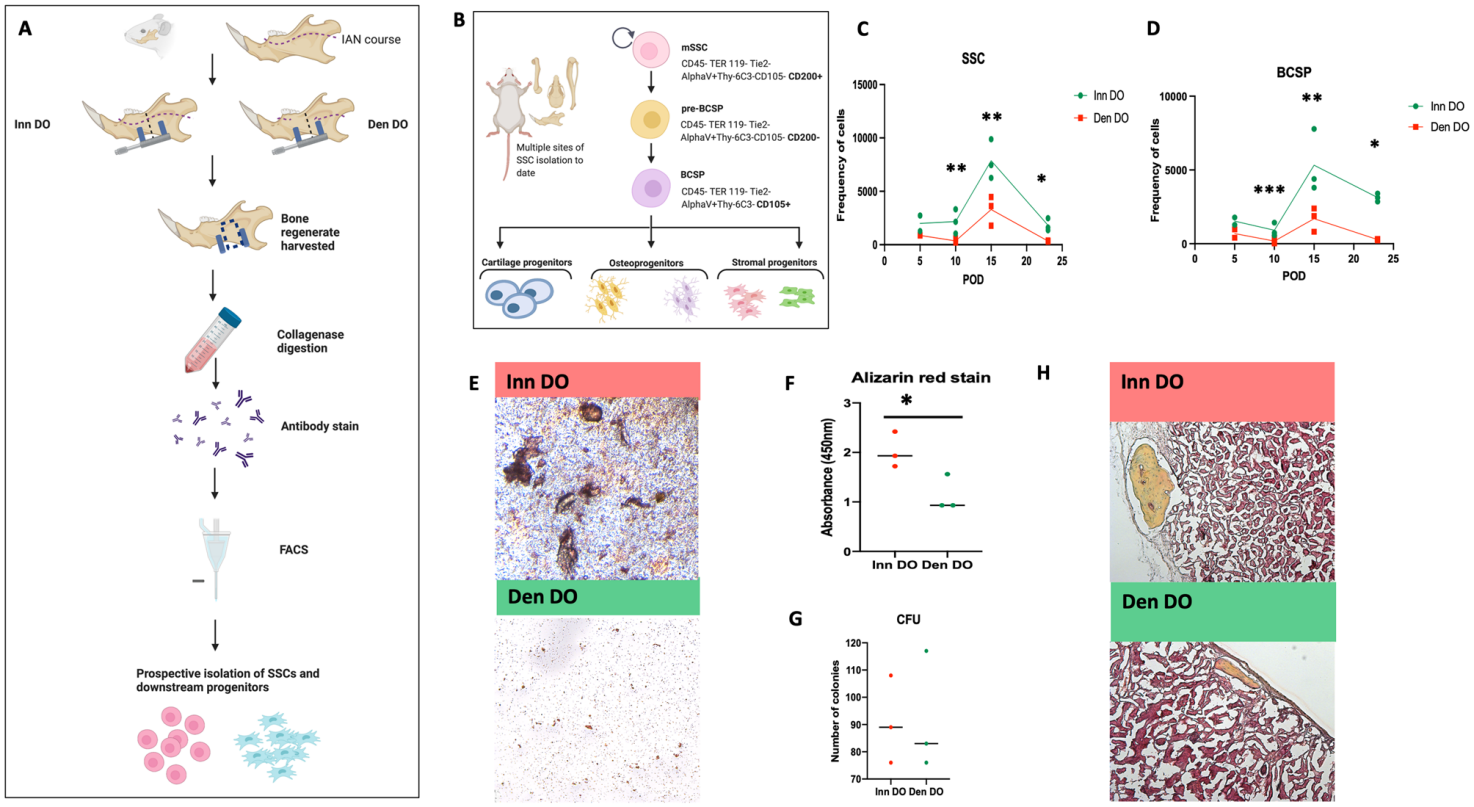
Mandible Skeletal Stem Cell Kinetics and Osteogenic Potential following DO are nerve dependent. (**A**) Experimental schematic: bone regenerate was harvested at multiple timepoints and cells were dissociated for FACS antibody staining and subsequent sorting / analysis. SSCs were then prospectively isolated for in vitro and in vivo analyses. Created with BioRender.com. (**B**) The mouse skeletal stem cell (SSC) is a single cell capable of giving rise to bone, cartilage and bone marrow stroma^14^ and has previously been isolated from long bone^14,15^, long bone fractures^17,18^, and mandibles^12,13^. The lineage tree with relevant immunophenotypes of SSC and downstream progenitors are shown. Here, *BCSP* bone, cartilage and stromal progenitor. Created with BioRender.com. (**C**) Frequency of SSCs isolated using FACS from Inn DO mandibles (red) versus Den DO mandibles (green) at multiple post-operative time-points: (left to right) POD 10, POD 15, POD 23. Here * denotes p < 0.05, ** denotes p < 0.01. (**D**) Frequency of BCSPs isolated using FACS from Inn DO mandibles (red) versus Den DO mandibles (green) at multiple post-operative time-points: (left to right) POD 10, POD 15, POD 23. Here * denotes p < 0.05, ** denotes p < 0.01, *** denotes p < 0.001. (**E**) Alizarin red staining of mSSCs isolated from Inn DO (red, above) versus Den DO (green, below) and cultured in osteogenic differentiation medium to examine their in vitro osteogenic potential. (**F**) Graphical representation of the intensity (absorbance wavelength 450 nm) of alizarin red stain at POD 15 of (red) Inn DO mSSCs versus (green) Den DO mSSCs. Here * denotes p < 0.05. (**G**) Graphical representation of the colony forming unit capacity of POD 15 (red) Inn DO mSSCs versus (green) Den DO mSSCs. (**H**) Histological representation of ossicles formed 6 weeks following kidney capsule transplantation assays^14^ (top, Inn DO versus bottom, Den DO) of 20,000 mSSCs isolated at POD 15.

Using fluorescence-activated cell sorting (FACS), we investigated nerve-dependent SSC kinetics to study their relative frequency at different points following DO in the presence or absence of IAN mandible innervation (Fig. 2A,B, Supplementary Fig. 2). Here, using age and sex-matched animals, Inn DO and Den DO mandibular regenerates were micro-dissected, harvested and prepared for FACS at multiple time-points following DO (POD 10, 15, 23) as previously described^15^. A significantly reduced frequency of SSCs was observed in Den DO relative to Inn DO at all time-points (Fig. 2C). In addition, profiling of the downstream “bone-cartilage-stromal progenitor” also demonstrated a significant lack of expansion in the Den DO relative to Inn DO regenerates at POD 10, 15, 23 (Fig. 2D).

We next questioned whether IAN denervation also resulted in altered osteogenic potential relative to control innervated mandibles. Following prospective isolation using FACS, SSCs from Den DO and Inn DO mandible regenerates at POD 15 underwent an in vitro osteogenic differentiation assay, which demonstrated significantly reduced Alizarin red staining in Den DO SSCs relative to Inn DO SSCs (Fig. 2E,F). There was, however, no difference observed in the colony forming capacity of the Den DO SSCs relative to Inn DO SSCs (Fig. 2G). We then proceeded to an in vivo assay to examine intrinsic osteogenic capacity of SSCs. SSCs were transplanted beneath the kidney capsule in Nod-Scid gamma mice^19,20^ and harvested 6 weeks later. Inn DO SSC transplantation resulted in larger bone ossicles relative to an equal number of transplanted Den Do SSC, demonstrating superior in vivo osteogenesis in SSCs which were harvested from innervated distraction regenerate (Fig. 2H).

### Single cell RNA sequencing demonstrates IAN innervation affects SSC transcriptional priming

**Figure 3:**
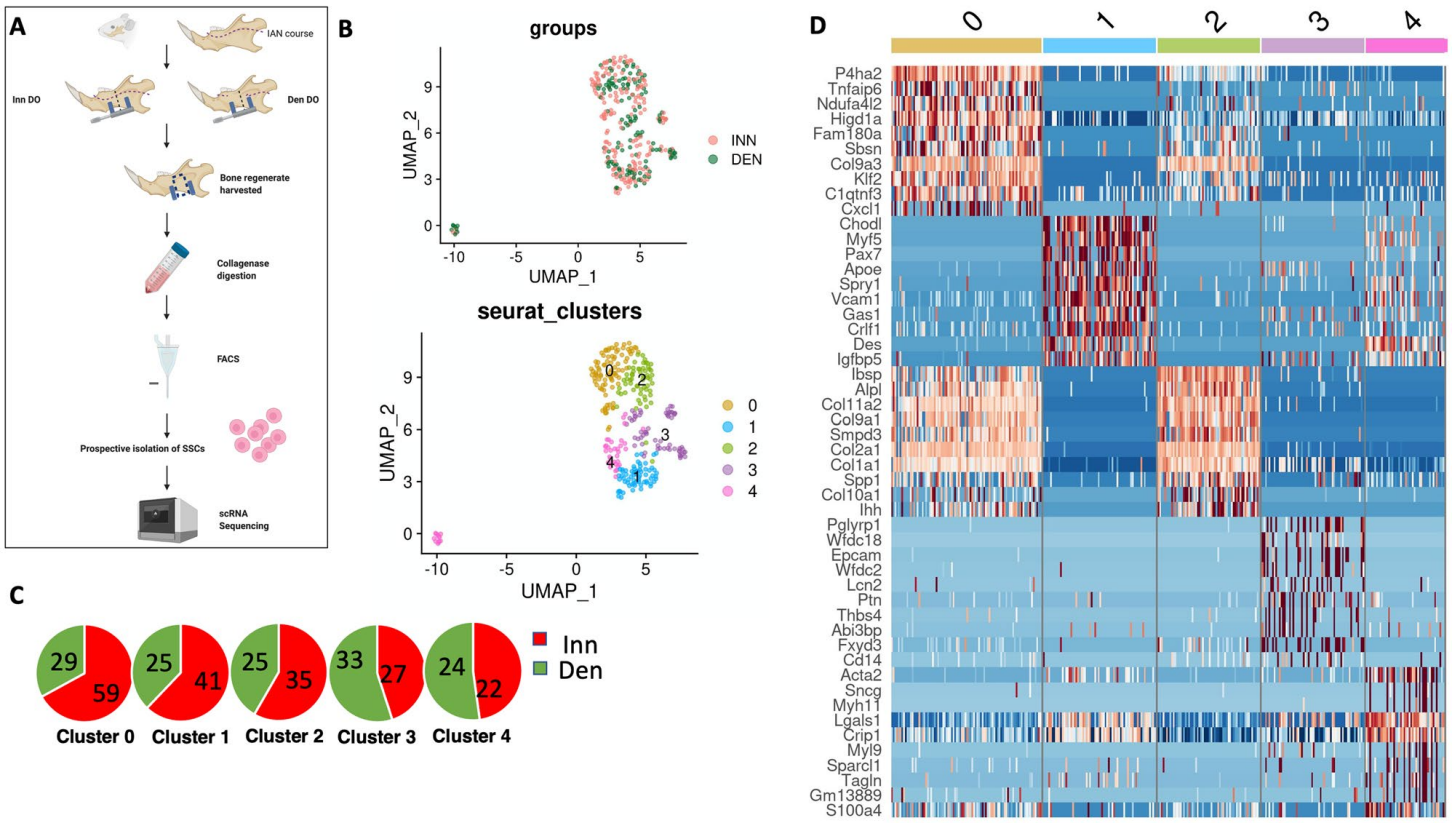
Single Cell RNA Sequencing Demonstrates Mandible IAN Innervation Affects SSC Transcriptional Priming. (**A**) Experimental schematic: Den DO and Inn DO mSSCs were isolated from mandible distraction regenerate at POD 15 and underwent single cell RNA sequencing using the 10X genomic platform. Created with BioRender.com. (**B**) Uniform manifold approximation and projection (UMAP) plot showing scRNA-seq data from (top) Inn DO (red) and Den DO (green) FACS-isolated mSSCs at POD 15. (Bottom) Five unique clusters of mSSCs are identified. Colors as labeled in the figure panel. (**C**) Pie chart demonstrating relative breakdown of representation of Inn DO mSSCs (red) versus Den DO mSSCs (green) in each cluster (left to right: 0, 1, 2, 3, 4). (**D**) Heat map representing top 10 differentially expressed genes characteristic to each of the five transcriptionally-defined clusters. Red: upregulation; blue: downregulation.

SSCs were then prospectively isolated using FACS for single cell RNA sequencing (scRNAseq) analysis using the 10X Genomics platform (Fig. 3A) at POD 15. Partitional clustering identified five SSC clusters (Fig. 3B)^21^. Cluster 0, 1 and 2 had a predominance of Inn DO SSCs relative to Den DO SSCs, whereas Cluster 3, 4 were slightly increased in Den DO SSCs relative to Inn DO SSCs (Fig. 3C). We then performed gene set enrichment analysis using EnrichR Database to identify expression programs associated with each cluster (Fig. 3D, Supplementary Fig. 3A–D). Cluster 0 was defined by genes such as *Collagen 9*, *P4ha2, and Chad,* and pathway analysis of the top 100 genes demonstrated enrichment of pathways such as ossification and cell differentiation. Cluster 1 was defined by genes such as *Chodl, Crlf1, Myf5* and *Pax7* which promote development of the nervous system, survival of neuronal cells, neural crest development and stem cell proliferation respectively. Cluster 2 was defined by genes such as *Ibsp*, *Col1a1*, *Col2a1*, *Spp1* and *Col10a1,* which are upregulated in osteogenesis. Contrastingly, pathway analysis of Clusters 3, 4 demonstrated enrichment of genes associated with focal adhesion kinase (FAK) including *Col1a1*, *Col1a2, Itgb5, Col5A1, TNN* and *Col1a1*, *Col3a1, Col1a2, Ibsp, Col6a2, Col5a2, Spp1, respectively.* Using chromatin and transcriptional profiling, Ransom and colleagues previously demonstrated that SSCs gain accessibility within the FAK signaling pathway during mandibular distraction and that mechanotransduction via FAK in SSCs activates a gene-regulatory program that drives neural crest cell-like reversion of SSCs undergoing distraction, facilitating stem cell driven regeneration of adult skeletal tissue^13^. Taken together, these data suggest that, at this early timepoint, distraction in the setting of denervation leads to a relative difference in osteogenesis and mechanotransduction when compared to distraction in the setting of innervation.

### Reduced osteogenesis evident in a pediatric patient undergoing bilateral mandibular DO in the setting of unilateral IAN denervation

**Figure 4:**
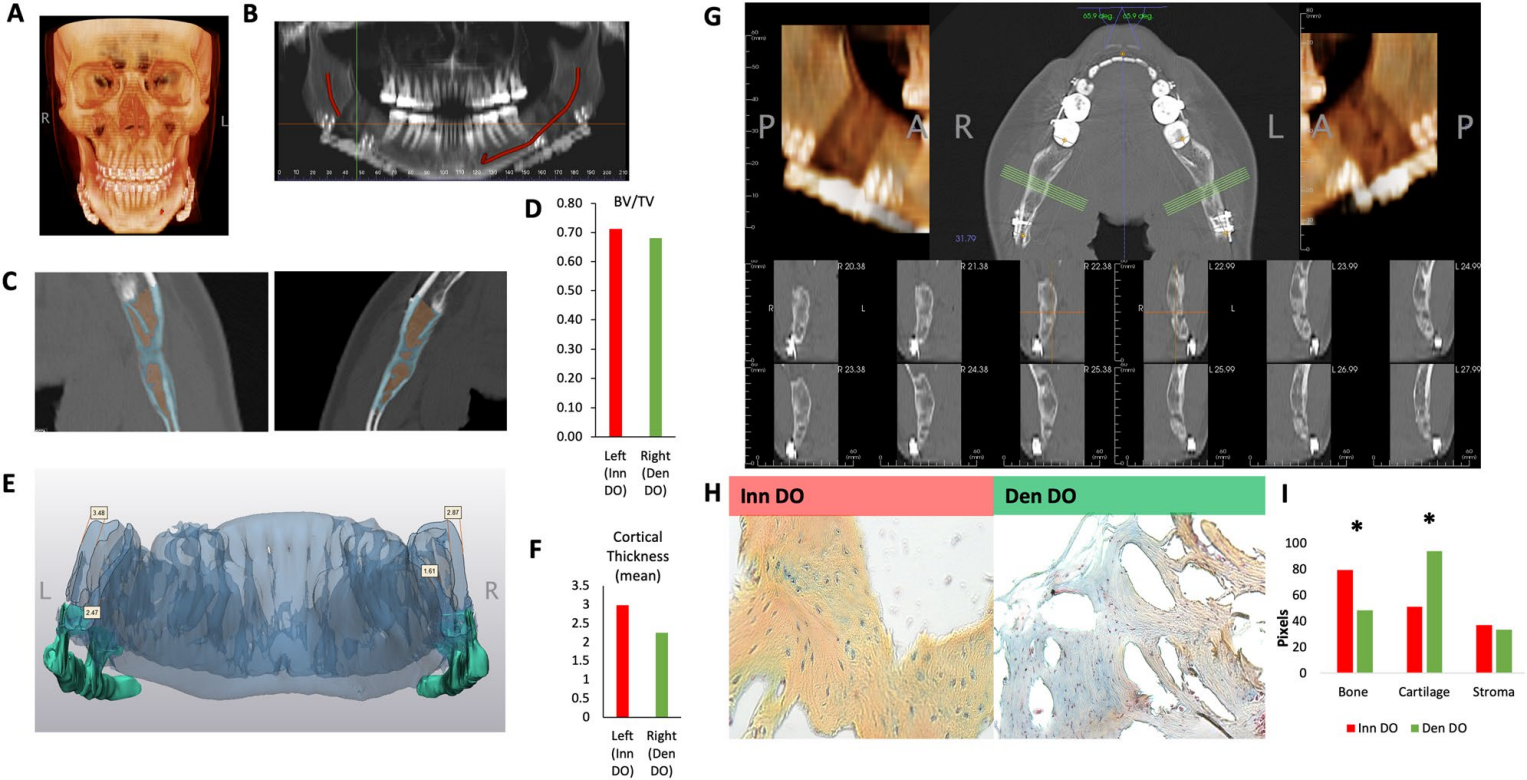
Reduced Bone Density Post Bilateral Mandibular DO Evident in Hemi-mandible of a Patient with IAN Injury Relative to Hemi-mandible with Intact IAN. (**A**) Coronal image of Panorex CT of 13 year old female prior to distractor removal at 3 months post-distraction. (**B**) Representative image of clinical status and course of IAN coursing through mandible at 3 months post distraction. Right: IAN clinically absent, Left: IAN clinically intact. (**C**) (Left) Antero-posterior image of mandible at site of distraction in left hemi-mandible with clinically intact IAN at 3 months post distraction. (Right) Antero-posterior image of mandible at site of distraction in right hemi-mandible with clinically absent IAN at 3 months post distraction. (**D**) Graph demonstrating analysis of bone regenerate callus mineralized volume fraction (bone volume/total tissue volume, or BV/TV) of Inn DO (red) versus Den DO (green) as determined by CT histomorphometry. (**E**) 3D reconstruction of mandible (oriented to illustrate the lingual mandible) at 3 months post distraction demonstrating cortical thickness as determined by CT analysis. (**F**) Graph demonstrating mean cortical thickness of distraction regenerate of Inn DO (red) versus Den DO (green) as determined by CT analysis. (**G**) (Above) Left: Reconstructed CT image demonstrating bone distraction regenerate on denervated side, Middle: Axial image of distracted mandible with green lines representing multiple sites of analysis, Right: Reconstructed CT image demonstrating bone distraction regenerate on innervated side. (Bottom): Multiple representative coronal slices of right (left 3 images top and bottom) Den DO vs left Inn DO (right 3 images above and below) distraction regenerate mandible at 3 months post-distraction. (**H**) Movat’s pentachrome stained representative sections of Inn DO (red) vs Den DO (green) mandible at 3 months post-distraction. Here, yellow denotes bone formation, blue denotes cartilage formation, purple denotes fibrous stroma formation. (**I**) Graph demonstrating composition of human distraction regenerate (bone, cartilage or fibrous stroma). There is significantly greater bone present in Inn DO (red) relative to Den DO (green) (*p < 0.05) and significantly greater cartilage present in Den DO relative to Inn DO (*p < 0.05).

A 13 year-old girl with a past medical history of micrognathia underwent bilateral mandibular DO and was noted post-operatively to have a new unilateral (right) IAN clinical deficit (defined by reduced sensation in the anatomical distribution of the IAN, the lower lip) with a contralateral clinically intact (left) IAN nerve (normal lower lip sensation) (Fig. 4A,B). Panorex CT prior to distractor removal at 3 months post-distraction demonstrated the presence of adequate bone on each side. However, acknowledging the limitation of a single human specimen, there was reduced Bone Volume/Tissue Volume and reduced mean cortical thickness of the bony regenerate of the denervated right relative to the left mandible with the IAN intact when analyzed radiologically (Fig. 4C–G). At the time of the distractor removal following 11 mm of advancement and consolidation, the patient continued to have reduced sensation in the distribution of the IAN and we, thus, obtained a small specimen of the bilateral hemimandibles for histological and FACS analysis to determine if there was any difference in the mandible regenerate in the setting of intact versus deficient sensory innervation via the IAN. Using Movat’s Pentachrome staining, we first observed an increase in cartilage presence (blue staining) relative to bone presence (yellow staining) in the bony regenerate in Den DO relative to Inn DO mandible regenerate (Fig. 4H,I). Due to the limitation of a single patient specimen, radiological and histological comparisons were not amenable to statistical analysis.

**Figure 5:**
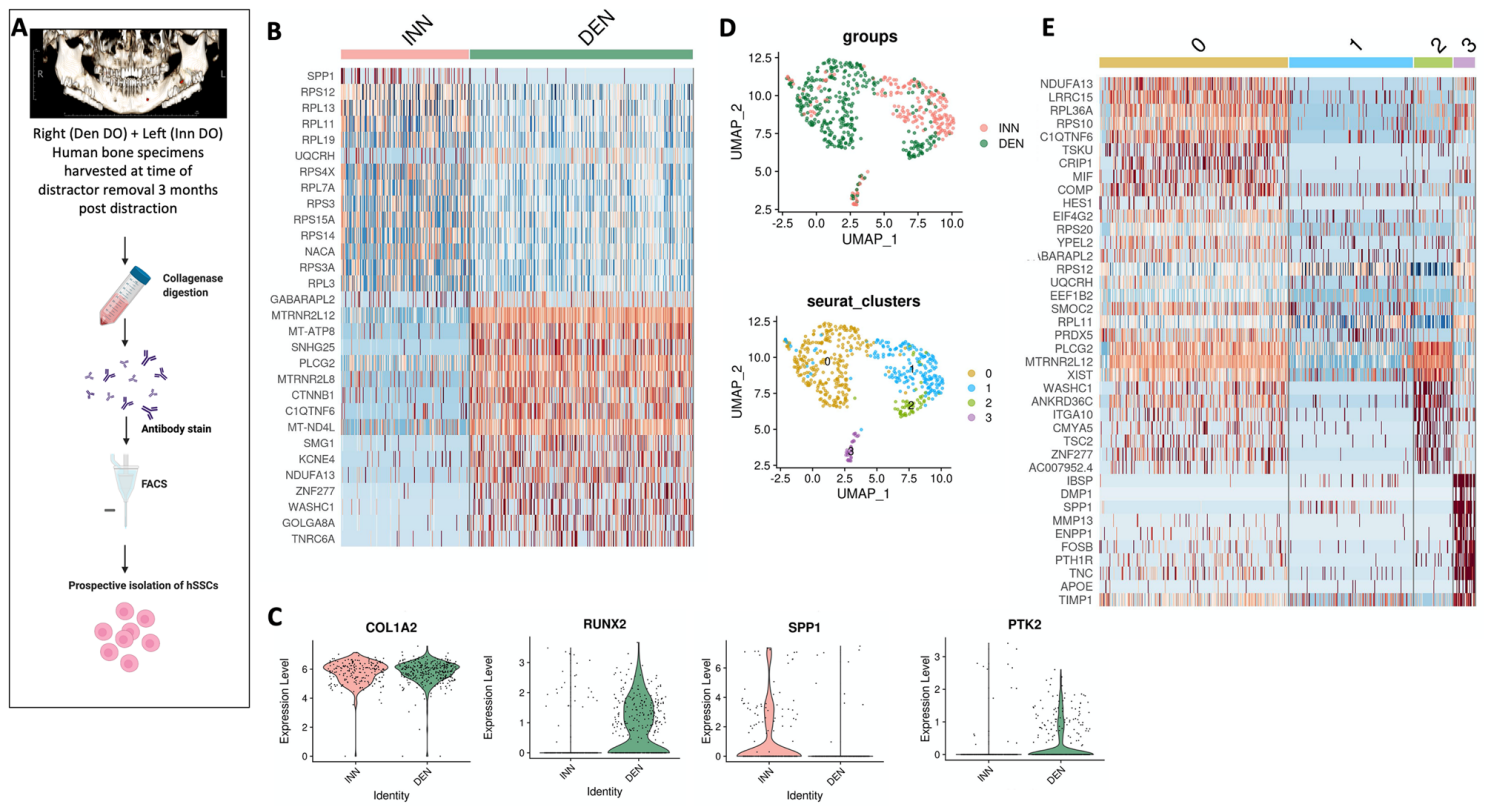
Human Mandible Distraction Regenerate scRNA Sequencing analysis. (**A**) Experimental schematic: hSSCs were prospectively isolated using FACS from mandible distraction regenerate at 3 months post distraction from bilateral hemi-mandibles: left with clinically intact IAN (Inn DO) and right with clinically absent IAN (Den DO) and underwent scRNA seq using the 10X genomic platform. Created with BioRender.com. (**B**) Heat map representing top differentially expressed genes characteristic of Inn DO and Den DO hSSCs. Red: upregulation; blue: downregulation. (**C**) Violin plots demonstrating transcriptional expression of left to right: COL1A2, RUNX2, SPP1, PTK2 in Inn DO hSSC (red) and Den DO hSSC (green). (**D**) Uniform manifold approximation and projection (UMAP) plot showing scRNA-seq data from (top) Inn DO (red) and Den DO (green) FACS-isolated hSSCs three months post distraction (Bottom) Four unique clusters of hSSCs are identified. Colors as labeled in the figure panel. (**E**) Heat map representing top differentially expressed genes characteristic of the four unique clusters of hSSCs. Red: upregulation; blue: downregulation.

Following collagenase digestion of the remaining bony regenerate specimen, we next set out to prospectively isolate human SSCs (hSSCs)^22^ using FACS (Fig. 5A). The hSSC is a self-renewing and multipotent SSC, present in fetal and adult bones, that generates progenitors of bone, cartilage and stroma and undergoes local expansion in response to skeletal injury (Supplementary Fig. 4)^22^. Given the radiological and histological differences seen in Den DO versus Inn DO mandible regenerate, we questioned if there would be transcriptional heterogeneity between hSSCs determined by the presence or absence of IAN during mandibular DO, as we saw in mice. Here hSSCs were isolated using FACS and prepared for single cell RNA sequencing (scRNAseq) analysis using the 10X Genomics platform. Comparing Inn DO versus Den DO hSSCs, we observed increased *Osteopontin* (*SPP1)* expression in Inn DO hSSCs relative to Den DO hSSCs, in addition to increased ribosomal protein transcription (Fig. 5B). Furthermore, there was notable differential transcription of *RUNX2* and *PTK2* between Den DO hSSC and Inn DO hSSC, with increased RUNX2 and PTK (and reduced SPP1) present in the Den DO hSSC at this late stage of consolidation relative to Inn DO hSSC (Fig. 5C). RUNX2 is the furthest upstream transcription factor in the regulation of osteoblast differentiation^23,24^, whereas *SPP1* is a late marker of osteogenic differentiation^25^, and ribosome biogenesis is associated with differentiation during skeletal development, playing a critical role in intramembranous ossification of the neural-crest derived craniofacial skeleton^26^. *PTK2* is the gene responsible for the translation of Focal adhesion kinase 1 protein, a key pathway component for mechanotransduction. These findings suggest that the delayed osteogenesis seen in denervation may be mediated by altered mechanotransduction in the human mandible, mirroring that seen in the mouse model.

Furthermore, PCA of the isolated hSSCs revealed the presence of four distinct transcriptional clusters (Fig. 5D). Cluster 0, 2 have an increased representation of Den DO hSSCs relative to Inn DO hSSCs; whereas Cluster 1 is dominated by Inn DO SSCs relative to Den DO SSCs. We then performed gene set enrichment analysis using EnrichR Database to identify expression programs associated with each cluster (Supplementary Figs. 5, 6). On pathway analysis, Cluster 0 is associated with extracellular matrix organization, TGFb regulation of extracellular matrix and Collagen biosynthesis. Cluster 1 is associated with pathways such as BDNF signaling, in addition to cellular adhesion signaling. Cluster 2 is associated with pathways including YAP and TAZ gene expression as occurs in mechanotransduction, messenger RNA splicing, and collagen binding. Cluster 3 is associated with pathways associated with cytoplasmic ribosomal proteins, BDNF signaling, and bone mineralization regulation (Fig. 5E).

### Exogenous application of fibromodulin improves osteogenic potential of denervated SSCs in vitro

**Figure 6:**
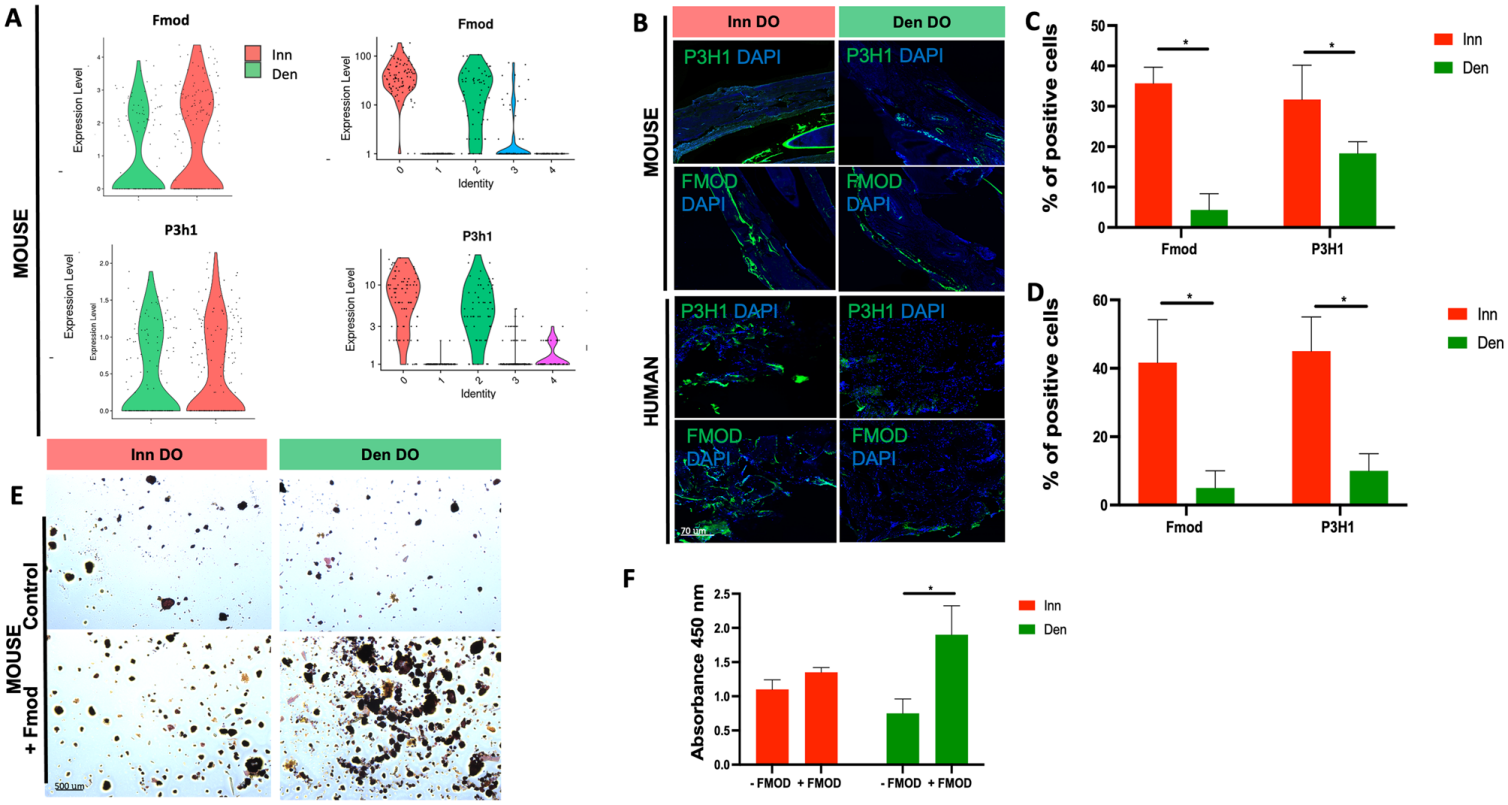
Fibromodulin Expression is Increased in Innervated Distraction and Exogenous Application Improves Osteogenic Potential In Vitro In Den DO. (**A**) Violin plots of FMOD and P3H1 in (left) Inn DO and Den DO mSSCs and (right) mSSC pro-osteogenic clusters. (**B**) Immunohistochemistry (IHC) analysis of P3H1 and Fmod expression Inn DO and Den DO human (bottom) and mouse (top) tissue specimens. (Blue, DAPI). (n = 3). (**C**) Quantification of FMOD (left) and P3H1 (right) IHC staining of mouse specimens. Red = Inn DO mSSC, green = Den DO mSSC. (**D**) Quantification of FMOD (left) and P3H1 (right) IHC staining of human specimens. Red = Inn DO mSSC, green = Den DO mSSC. (**E**) Alizarin red staining of Fmod treated Den Do and Inn DO mSSCs at postoperative day 14. (**F**) Graph demonstrating quantification of alizarin red staining following Fmod treatment (−FMOD = control, +FMOD = treatment group). p * < 0.05. Red = Inn DO mSSC, green = Den DO mSSC. (n = 2).

Analysis of the mSSC clusters (which had a predominance of innervated relative to denervated SSCs) demonstrated that FMOD and Prolyl-3-hydroxylase-1 (P3H1) transcriptional expression was reduced in denervated relative to innervated mouse mandible regenerate (Fig. 6A). This prompted us to assess FMOD and P3H1 expression using immunohistochemistry in both human and mouse denervated versus innervated bone regenerate. Here, we saw significantly reduced expression of FMOD and P3H1 in both human and mouse denervated versus innervated bone regenerate (Fig. 6B–D, *p < 0.05**)**. Exogenous FMOD was then added to mouse DO-Den and DO-Inn SSCs undergoing in vitro osteogenic differentiation, resulting in increased osteogenic potential of DO-Den SSCs in comparison to control untreated DO-Den SSCs, modeling the superior osteogenic potential of DO-Inn SSCs (Fig. 6E,F, *p < 0.05**)**. We were unable to also perform this assay with hSSCs as we were limited by the amount of tissue that we could obtain from the bone regenerate.

## Discussion

In this study, we identify that denervation preceding DO results in impaired osteogenesis and reduced SSC amplification and osteogenic potential in mice. Intriguingly, a rare human patient specimen was also analyzed, and resultant data suggested that histological, radiological, and transcriptional alterations seen in mouse DO may be conserved in the setting of denervated human mandible distraction three months following distraction. This led us to question whether this finding was also conserved in mice at a later time point and thus, osteogenesis was examined radiologically at ninety days following distraction in mice in the setting of an innervated or denervated mandible. Again, we determined that osteogenesis was reduced even in mice at POD 90 in the setting of denervation, thus the denervated mandible was not able to “catch up” with respect to osteogenesis.

Previous work by Ransom and colleagues demonstrated that mandibular distraction led to activation of mouse skeletal stem cells. Given our finding of impaired osteogenesis in the setting of denervation, one questions where do skeletal stem cells reside? In 1987, Brockes reported that blastemal cells can fully regenerate an amphibian limb in the presence of nerve axons at the amputation plate^27^. In bone, sensory nerve fibers innervate the periosteum. Kojimoto and colleagues showed that when periosteum was removed during distraction osteogenesis^28^, bone regeneration was markedly disturbed—one could question that this could be secondary to a lack of innervation, vascularity, or due to a reduction in a stem cell repository. Our study suggests, of the three possibilities put forth by Kojimoto et al.^28^, that periosteal innervation affects SSC activation and the intrinsic osteogenic potential of SSCs.

Recent literature suggests that the paracrine role of nerves has been underestimated for decades. Rather than simply facilitating the transmission of sensory stimuli to the cortical homunculus or facilitating the transmission of motor stimuli from the motor cortex, the importance of nerves in aiding tissue response to injury is now beginning to come to light^12,29^. In the developing mouse skeleton, tropomyosin receptor kinase A (TrkA)-expressing sensory neurons home to long bone periosteum at sites of nerve growth factor expression, resulting in expansion and activation of skeletal progenitors^30^. In fact, inactivation of TrkA signaling during embryogenesis leads not only to impaired innervation, but also to decreased numbers of Osterix-expressing bone progenitors and decreased femoral length and volume^30^. Similarly, Li and colleagues also demonstrated that temporal inhibition of TrkA led to reduced sensory fibers and revascularization, together with delayed ossification of fracture callus^31^. Lee et al. defined the regulatory role of neural input in a well characterized trauma-induced model of heterotopic ossification, whereby they demonstrated that surgical denervation led to abrogated endochondral bone formation which was thought to occur following a shift from TGF-B to FGF signaling activation amongst chondroblasts^29^. Furthermore, our laboratory has previously demonstrated that mouse SSCs rely on paracrine factors (OSM, PTH and PGFRA) secreted by Schwann cells to enable mandible unicortical fracture repair^12^, however these factors were not implicated in DO. To our knowledge, this study is the first to demonstrate the significant role of innervation in DO in both mouse and human mandible.

Single cell RNA sequencing analysis of mouse SSCs at POD 15 revealed that there was a predominance of innervated mSSCs in clusters dominated by pathways related to bone formation, whereas clusters related to mechanotransduction signaling were increased in denervated mSSCs. In our rare human patient specimen, we demonstrated that there was notable differential transcription of *Osteopontin*, *RUNX2,* and *PTK2* between Den DO hSSC and Inn DO hSSC, suggesting that mandible denervation prior to DO results in delayed osteogenesis via mechanotransduction during distraction (as demonstrated by increased relative expression of *RUNX2* and *PTK2* in Den DO), in comparison to a relative increase in *Osteopontin* transcription in Inn DO. Mechanotransduction has been shown to mediate DO, and thus, the observation that increased transcriptional activation of mechanotransduction signaling is seen in Den DO is perplexing. It has, however, been hypothesized that mechanotransduction stimuli may be facilitated by nerves in muscle^32^, and one could hypothesize that perhaps denervation leads to delayed propagation of stretch stimuli that mediate mechanotransduction, and thus, leads to delayed DO.

FMOD expression together with P3H1 were then noted to be reduced in Den DO relative to Inn DO mandible mSSCs. FMOD is a non-collagenous small leucine-rich repeat protein which is present in the skeletal extracellular matrix and plays a role in cartilage fibrillogenesis^33^, muscle development^34^, angiogenesis^35,36^, wound healing^37^ and tumorigenesis^38^. FMOD has been shown to activate lysyl oxidase which catalyzes oxidative deamination of lysine residues in extracellular matrix collagen, which facilitates collagen cross-linking, important for optimal bone formation and strength^39^. It has been detected in embryonic sclerotomes at 9.5 days post coitus^40^ and is expressed by fetal chondrocytes and osteoblasts during endochondral and intramembranous ossification^41^. In addition, FMOD has been shown to reprogram dermal fibroblasts to a multipotent state with the potential for osteogenic lineage differentiation without gene transduction^42,43^ and to improve wound healing, reducing scar size, increasing tensile strength, and improving dermal collagen architecture organization, when applied intra-dermally to wounds of normal and mechanically loaded red Duroc pig wound models^44^. P3H1 is a resident enzyme in the endoplasmic reticulum which modifies collagen post-translationally. Given these important developmental and regenerative pathways, we then assayed protein expression of FMOD and P3H1 in mouse and human DO-Inn and DO-Den bone regenerate and interestingly, saw reduced expression of FMOD and P3H1 in the setting of DO-Den relative to DO-Inn. Exogenous FMOD application rescued the osteogenic potential of denervated DO mSSCs in in vitro osteogenic differentiation assays, demonstrating a potential link between denervation and FMOD expression.

Our study is limited by the availability of a single rare human mandible DO bone regenerate specimen with a unilateral clinically intact IAN and a contralateral clinically deficient IAN, and thus we are unable to draw definitive conclusions from the human data. While the human mandible distraction specimens were obtained from the new bone formed by distraction bilaterally, the sampling of tissue from a single site on each mandible may also be altered due to possibility of spatial variability in the regenerate. In addition, while we had access to multiple time-points for our small animal studies, we are limited by a single time-point of the rare human specimen (three months post distraction). A further limitation is that a denervation injury may lead to local inflammation at the time of the distractor placement.

While further work certainly remains to be completed, these results lead us to question how they might translate to improvement in patient care. DO is a lengthy and cumbersome process for patients and their families, and can be complicated by post-operative skeletal relapse, whereby the final length of bone diminishes after treatment. If we can better understand the mechanisms driving DO, we can better treat children born with congenital skeletal anomalies and adults with acquired skeletal deficiency and improve health-related quality of life. Perhaps, in the setting of a denervation injury of the mandible at the time of distractor placement and osteotomy, one could argue for the provision of further time for consolidation prior to removal of distractors relative to an innervated mandible. In addition, as DO is a complex and lengthy process with significant burden on our patients and their families, our data prompt an examination of how exploitation of nerve pathway signaling may expedite osteogenesis and thus, shorten treatment courses for our vulnerable patient population.

## Materials and methods

### Animals

All animal experiments were performed in accordance with the Stanford University Animal Care and Use Committee guidelines and reported in compliance with the Arrive guidelines. Ten-week-old male C57BL/6 J mice *(Jackson Laboratories, Maine, USA)* underwent gradual distraction with nerve intact and gradual distraction following either a segmental defect in the inferior alveolar nerve (DO Den) or dissection and preservation of the inferior alveolar nerve (DO Inn) (see below). Distraction was performed using our laboratory’s established mouse mandibular distraction protocol^13^. Thy1-YFP homozygous mice were used to validate the model of inferior alveolar nerve (IAN) denervation. All experiments were carried out according with Stanford University Animal Care and Use Committee guidelines. All experimental protocols were approved by Stanford University Animal Care and Use Committee (Protocol APLAC 12765).

Animals were housed in cages of up to five littermates in temperature- and light- controlled environs and fed ad libitum.

### Mouse mandibular distraction model

This was performed using our laboratory’s established distraction model using the operating microscope^13^. Briefly, animals were anaesthetized (with 20 mg kg^−1^ Ketaset, 1.5 mg kg^−1^ xylazine and 0.2 mg kg^−1^ acepromazine maleate), given a preoperative dose of antibiotics (10 mg kg^−1^ cefazolin) and prepped with Betadine, and their incisors were clipped. An incision was made over the right hemimandible, the masseter muscle divided, and the mandible exposed. One 0.6-mm hole was drilled 3 mm anterior, and one 3 mm posterior, to a line dividing the mandibular ramus just posterior to the third molar. An osteotomy was then performed posterior to the third molar using a diamond disc saw under constant saline irrigation (*Brasseler*). Distraction plates were secured with insertion of tight-fit 0.65-mm screws (*McMaster-Carr*). The muscle and skin were then closed in layers. Animals tolerated the procedure well and received appropriate postoperative analgesia. Postoperative mortality was < 5%, and all deaths were replaced with new animals to obtain the final numbers. Mice were fed a soft diet (*Dietgel, Clear H20, Inc, Maine, USA*) for nutritional optimization. Subcutaneous boluses of normal saline were administered daily for 10 days post-operatively.

### Mandible denervation

This was performed using the operating microscope at a magnification of 20X *(Carl Zeiss, Jena, Germany).* Following drill hole placement, a unicortical mandibular osteotomy was performed. This allowed the osteotomy to be completed (bicortical) with scissors and forceps to ensure identification and preservation of the inferior alveolar neurovascular bundle which was identified coursing through the canal in the mandible. For IAN denervation, a segmental defect was then performed in the IAN (4 mm), being careful to preserve the vascular bundle coursing with the nerve. The IAN disruption model in the setting of distraction osteogenesis was validated using Thy1-YFP mice. Thy1-YFP mice underwent distraction osteogenesis with DO Inn or DO Den and were imaged stereomicroscopically at POD 43 and 90 to confirm IAN disruption due to the significantly decreased YFP expression in IAN denervated mice.

### Micro-computed tomography

At POD 43, devices were removed before fixation in 2% paraformaldehyde at 4 degrees Celsius. The mandibles were then scanned using the Bruker Skyscan 1276 with a source voltage of 85 kV, a source current of 200 μA, a filter setting of AI 1 mm, and pixel size of 12 μm at 2016 × 1344. Phantom targets provided by the manufacturer were used to calibrate instrument measurements. Reconstruction was performed using the NRecon software *(Bruker, MA, USA)* and 3D images were produced using CTVol *(Bruker, MA, USA)*. CT histomorphometry was measured using a standardized region of interest of 10 mm as created in DataViewer *(Bruker, MA, USA)* using the CTAn software *(Bruker, MA, USA).* Results were reported in terms of mineralized volume fraction, which is defined as bone volume divided by total tissue volume.

### Histology preparation

Whole mandible tissue specimens were micro-dissected and kept on ice. Dissected tissue samples were fixed in 2% paraformaldehyde (PFA) at 4 degrees Celsius overnight and washed with phosphate-buffered saline (PBS) the following day. The specimens were decalcified in 19% EDTA in PBS at 4 degrees Celsius for four weeks with a change of EDTA every 48 h. Specimens were dehydrated and embedded in paraffin and sectioned at 8 mm. Representative sections were stained with Movat’s modified pentachrome solution, which distinguishes the tissues as follows: bone appears yellow, cartilage appears blue-green, muscles appear bright red, and stroma appears brown. A minimum of six micro-dissected mandibles in each cohort/timepoint were prepared for histological examination. The samples were quantified for bone composition in pixels using the color deconvolution plug-in on the ImageJ software *(National Institutes of Health, Maryland, USA)*.

### Confocal imaging to validate denervation/innervation model

Following dissection, specimens were washed in phosphate buffered saline (PBS) three times and mounted on onto glass slides with DAPI Fluromount-G (0100-20; *SouthernBiotech, Birmingham, AL*). Confocal microscopy imaging was performed with a LSM 880 inverted confocal microscope (*Leica Microsystems, Wetzlar, Germany)* with the 20X objective) located in the Cell Sciences Imaging Facility *(Stanford University, Stanford, CA)*. Raw image stacks were into imported into ImageJ (*National Institutes of Health, Maryland, USA*) for analysis.

### FACS isolation of mouse skeletal stem cells from mandible bone regenerate

Mandible bone regenerate was microdissected (dissected anterior of condyle but posterior to third molar) and prepared for FACS as previously published by our laboratory^13,15^. In brief, mandible bone regenerates were serially digested in collagenase digestion buffer supplemented with DNase at 37 °C for 40 min under constant agitation; total dissociated cells were filtered through 40-mm nylon mesh, pelleted at 200 g at 4 °C, resuspended in staining medium (2% fetal calf serum (FCS) in PBS), and stained with fluorochrome-conjugated antibodies against CD45, Ter119, CD202b, Thy1.1, Thy 1.2, CD105, CD51 and 6C3, and with a streptavidin-conjugated antibody for CD200. Propidium iodide staining was performed to exclude dead cells. FACS analysis was performed on a FACS Aria II Instrument (BD Biosciences) using a 70-mm nozzle. Gating schemes were established with fluorescence-minus-one controls and propidium iodide was used for viability staining. All cell populations were double-sorted for purification and subsequently evaluated for their functional responses as outlined below. Flow-cytometry plots are representative of a minimum of three independent experiments (each assessing > / = five mice during each replicate). To calculate cell population frequencies, we micro-dissected bone regenerates using the operating microscope and evaluated a standard area of tissue (4 mm on either side of the distraction osteomy) and represent data as an average across at least three independent experiments. All molecular and flow-cytometric analyses of FACS-purified cell populations in the SSC hierarchy throughout this study were performed using double-sorted cells to ensure purity of each population.

### Cell culture

Skeletal Stem Cells were cultured in alpha-MEM GlutaMax supplemented with 10% Fetal Bovine and 1% Penicillin–Streptomycin (Gibco-Life Technologies, Grand Island, NY, USA) after seeding on gelatin coated wells. Cells were incubated under low O_2_ conditions (2% atmospheric oxygen, 7.5% CO_2_) for 48 h and then moved to standard conditions (5% CO_2_).

### Osteogenic differentiation assay

Upon isolation of cells by FACS, cells were cultured for over two weeks. Each cell-type condition (DO Inn / DO Den SSC) was then incubated with osteogenic medium for two weeks with the medium changed every other day. After undergoing two weeks of osteogenic differentiation, cells were washed with PBS followed by ultrapure water. The monolayer of cells was fixed with 100% ethanol for 15 min and stained with alizarin red solution for 1 h at room temperature. The cells were washed several times with ultrapure water and imaged for osteogenic potential immediately under a bright-field microscope. Following imaging, the cells were treated with a methanol/acetic acid mixture for 15 min and absorbance was detected with an Ultraspec 2100 UV/Visible Spectrophotometer *(Biochrom, Harvard Bioscience, UK)* at 450 nm to measure alizarin red concentration across each cell type.

### Colony forming capacity

Isolated DO Inn/DO Den mSSCs were directly plated onto precoated (0.1% gelatin) culture plates (100 cells per well in a 10 cm^2^ well plate) in MEMa medium with 20% FCS under low O2 (2% atmospheric oxygen, 7.5% CO_2_) conditions. Colony-forming units were identified using an inverted microscope under × 40 magnification. Specimens were examined under phase microscopy and a cloning ring was used for quantification. Colonies were assessed for size and cell morphology as previously described^14^. The cells were subsequently lifted for staining and analysis by FACS, or plated for tertiary colonies. SSCs were isolated from ten-week-old male mice according to strain, and colony-formation assays were performed in triplicate using a minimum of three biological replicates across three independent experiments (n = 9).

### Transcriptomic analysis

All RNA sequencing, bulk and single cell analyses were conducted by the Stanford Functional Genomics Facility (SFGF) core, at Stanford University *(Stanford, CA, USA)*.

### Single cell RNA sequencing (scRNA-seq)

Freshly isolated SSCs were double sorted using FACS for purity. Single cell RNAseq was performed via the Picelli method^45^. Lysis buffer plates were thawed on ice, then heated at 72 °C for 3 min in a Biorad C1000 Touch thermal cycler. First strand cDNA synthesis was performed in a 10 µl reaction with 100 Units of Clontech’s Smartscribe reverse transcriptase (CAT# 639538, CloneTEch, Mountain View, CA, USA), 10 Units RRI, 1X First Strand Buffer (CloneTEch, Mountain View, CA, USA), 5 mM DTT, 1 M Betaine (CAT# B0300-5VL, Sigma, St. Louis, MO, USA), 6 mM MgCl2, 1 uM Template Switch Oligo (TSO, (5′-AAGCAGTGGTATCAACGCAGAGTACATrGrG + G-3′, Exiqon/Qiagen, Hilden, Germany) at 42 °C for 90 min, 70 °C for 15 min. PCR pre-amplification was performed in a 25 µl reaction with 1X Kapa HiFi HotStart (CAT# KK2602, Kapa BioSystems, Wilmington, MA, USA), 0.1 uM ISPCR primer (5′-AAGCAGTGGTATCAACGCAGAGT-3′, Integrated DNA Technologies, Skokie, IL, USA) at 98 °C for 3 min, then 25 cycles of 98 °C for 20 s, 67 °C for 15 s, 72 °C for 6 min, then 72 °C for 5 min. Reactions were cleaned with SPRI beads on a Biomek FX and eluted in 25 µl water, 0.2 µl aliquots were run on an Fragment Analyzer High Sensitivity NGS 1–6000 kit. Barcoded sequencing libraries were made using the miniaturized Nextera XT protocol of Mora-Castilla^46^ in a total volume of 4 µl. Pooled libraries were sequenced on a Nextseq 500 High Output flow cell with 2 × 150 paired end reads.

### Transcriptomic data analysis

For scRNA-Seq analysis, the R package Seurat^47^ v2.3.0 was used for QC, analysis, and exploration of scRNA-Seq data using R v3.4.4. Gene counts were tabulated from STAR^48^ v2.5.3a mapping against mouse mm9 reference and used as input to Seurat. The counts coincide with those produced by htseq-count with default parameters. Genes expressed in at least 3 cells and cells with at least 200 expressed genes were retained for further analysis. Gene expression measurements for each cell were normalized by their total expression, scaled by 10,000, and log-transformed. Following normalization, genes that varied between single cells were identified. PCA was performed on genes to output a set of genes that most strongly defined a set of principal components. Seurat’s graph-based clustering approach was used to cluster the cells. Seurat was further used to perform UMAP clustering which placed cells with similar local neighborhoods in high-dimensional space together in low-dimensional space. Positive and negative markers in each cluster were identified by comparing genes in cells of one cluster against genes in all other cells. Only genes that were detected in at least 25% of cells in either of the two populations were tested. Violin plots were used to visualize expression of top markers in each cluster. Finally, heatmaps of the top marker genes across cells were plotted.

### Kidney capsule transplantation assay

SSCs or other skeletal cells were purified using FACS and resuspended in 2 μL of Matrigel (*Corning, New York, USA)* and then injected subcutaneously or underneath the renal capsule of 8–12-week-old immunodeficient NSG mice. Injected cells developed into a graft after 8 weeks. The grafts were surgically removed for analysis. This experiment was performed with three biological replicates.

### Human specimen harvest

Human distraction regenerate samples were obtained following removal of distractors under approved IRB protocol #28853. The small sample from the bilateral human mandible regenerate was sectioned into equal pieces using a razor blade under 2.5X loupe magnification after transferring directly from the operating room to the laboratory on ice. The tissue for histology was then placed into 2% PFA fixation. The regenerate for FACS analysis was then minced separately with razor blade, resuspended in 3000 U/mL type II collagenase (*Sigma-Aldrich, St Louis, MO, USA*) digestion buffer supplemented with 100 U/mL DNase I *(Worthington Biochemical Corporation, NJ, USA*) and incubated at 37 °C for 40 min under constant agitation. The supernatant was filtered through a 70 μm nylon mesh and quenched with staining media (2% fetal bovine serum (FBS), in phosphate-buffered saline (PBS), (*Gibco-Life Technologies, Grand Island, NY, USA*). Digestion was repeated twice more prior to centrifugation at 200 *g* at 4 °C followed by resuspension in staining media.

### FACS isolation of human skeletal stem cells

Human skeletal cells were separated from red blood cells (RBCs) and bone dust by density gradient separation using 1:1 Histopaque of density 1.119 g/mL (*Sigma-Aldrich, St Louis, MO, USA*). The buffy coat was collected, washed with staining media, and the resulting cell suspension was depleted of CD45 + cells by magnetic-activated cell sorting (MACS) (Miltenyi, Cat#130–045-801). Cells were blocked with mouse IgG and stained with fluorochrome-conjugated antibodies against CD45 (BioLegend, Cat#304029-BL), CD235a (BioLegend, Cat#306612-BL), CD31 (Thermo Fisher Scientific™, Cat#13–0319-82), CD202b (Tie-2) (BioLegend, Cat#334204), CD146 (BioLegend, Cat#342010), PDPN (Thermo Fisher Scientific™, Cat#17–9381-42), CD90 (THY1; BioLegend, Cat#328110), CD164 (BioLegend, Cat#324808), and CD73 (BioLegend, Cat#344016). Flow cytometry was performed on FACS Aria II (BD Biosciences). Gating schemes were established with fluorescence-minus-one (FMO: staining with all fluorophores except one) controls and negative propidium iodide (PI) (Sigma-Aldrich, Cat#P4170) staining (1 μg/ml) was used as a measure for cell viability.

### Exogenous FMOD ODM assay

FACS-isolated mSSC were harvested from DO Den and DO Inn specimens at POD 10 and cultured in 96 well plates over a course of 10 days in osteogenic medium with media changes every 48 h in duplicates. Cells were treated with and without a single dose of hFibromodulin (FMOD) protein (0.1 mg/ml, R&D 9840-FM-050) at 12 h to evaluate the ability of FMOD to augment osteogenic potential. The monolayer of cells was fixed with 100% ethanol for 15 min and stained with alizarin red solution for 1 h at room temperature. The cells were washed several times with ultrapure water and imaged for osteogenic potential immediately. Following imaging, the cells were treated with a methanol/acetic acid mixture for 15 min and absorbance was detected at 450 nm using NanoDrop™ 0ne^c^ Microvolume UV–Vis Spectrophotometer (ThermoFisher)^12^.

### Statistical analysis

Statistical analysis was performed using Graph Pad Prism *(California, USA)*. A one-way ANOVA with multiple comparisons was performed for the histological analysis. Unpaired t-tests were performed for the radiological analyses. Results are shown in brackets as mean +/− SD unless otherwise stated.

## Supplementary Information

Supplementary Figures.

## Data Availability

All data are available in the main text or the supplementary materials.
