# Supplementary material for: Denervation during mandibular distraction osteogenesis results in impaired bone formation
Source: Sci Rep. 2023 Feb 6;13:2097. doi: 10.1038/s41598-023-27921-9 (PMC9902545; doi:10.1038/s41598-023-27921-9)
Supplement: Supplementary file 1 — Supplementary Figures. [file 41598_2023_27921_MOESM1_ESM.pdf]

Supplementary Data:

Denervation During Mandibular Distraction Osteogenesis Results In Impaired Bone Formation

Ruth Tevlin MB BAO BCh, MRCSI, MD[1-3], Michelle Griffin MBChB MRCS PhD[1,3], Kellen Chen PhD[3,7], Michael Januszyk MD PhD[1,3], Nick Guardino BS[3], Amanda Spielman BA[3], Shannon Walters BS[4], Garry Evan Gold MD[4], Charles K.F. Chan PhD[5,6], Geoffrey C. Gurtner MD[7], Derrick C. Wan, MD FACS*[1,3] and Michael T. Longaker, MD MBA FACS*[1,3,5,6].

[1]*Division of Plastic and Reconstructive Surgery, Stanford Hospital and Clinics, Stanford, CA*

[2]*School of Postgraduate Studies, Royal College of Surgeons in Ireland, Dublin, Ireland.*

[3]*Hagey Laboratory for Pediatric Regenerative Medicine, Department of Surgery, Stanford University School of Medicine, Stanford, CA, USA*

[4]*Department of Radiology, Stanford University School of Medicine, 1201 Welch Road P263, Stanford, CA 94305*

[5]*Institute for Stem Cell Biology and Regenerative Medicine, Stanford University School of Medicine, Stanford, CA, USA.*

[6]*School of Medicine, Stanford University, CA*

[7]*University of Arizona Health Sciences, Department of Surgery, Tucson, Arizona*

**Corresponding authors of equal contribution*

Supplementary Figure 1. Validation of Denervation injury model and POD 90 micro-CT reconstructions and analyses.

A. Representative confocal microscopy of Thy1 (GFP) staining in denervated and innervated at POD 90. Scale bar = 200um
B. Graphs demonstrating (left) bone volume (BV) and (right) tissue volume (TV) at POD 30 and POD 43 respectively.
C. Representative μCT reconstructions of Inn DO (top) vs Den DO (bottom) mandibles at POD 90.
D. Graph demonstrating analysis of POD 90 bone regenerate callus mineralized volume fraction (bone volume / total tissue volume, or BV/TV) of Inn DO (red circles) versus Den DO (green circles) as determined by μCT histomorphometry.

Supplementary Figure 2. FACS gating strategy for mSSC in DO Den and DO Inn Mandibles.

Supplementary Figure 3. Mouse Mandible Distraction Regenerate scRNA Cluster Pathway and Gene Ontology analyses

A. (Below) EnrichR gene set enrichment analyses with graphs demonstrating pathways (determined by Wiki Pathways) associated with top 100 feature genes from each cluster 0, 1, 2, 3, 4 ranked by odds ratio.
B. EnrichR gene set enrichment analyses with graphs demonstrating gene ontologies (determined by GO BP) associated with top 100 feature genes from each cluster 0, 1, 2, 3, 4 as per p value ranking.
C. Heat map representing top differentially expressed genes characteristic of Inn DO and Den DO mSSCs. Red: upregulation; blue: downregulation.
D. (Top, Left) Uniform manifold approximation and projection (UMAP) plot showing scRNA-seq data from (top) Inn DO (red) and Den DO (green) FACS-isolated mSSCs at POD 15. (Top, Right) Five unique clusters of mSSCs are identified. Colors as labeled in the figure panel. (Middle) Feature plots depicting expression of key genes implicated in osteogenesis by mSSCs (left to right, top to bottom: Col1a1, Col1a2, Runx2, Atf4, Sp7, Spp1, Ihh, Ibsp, Pth1r). (Bottom) Feature plots depicting expression of key genes implicated in mechanotransduction by mSSCs (left to right, top to bottom: Ptk2, Taz, Jun, Yap1, Fos).

Supplementary Figure 4. Human Mandible Skeletal Stem Cell FACS Gating Strategy in DO Den (bottom) and DO Inn (top) Human Mandibles.

Supplementary Figure 5. Human Mandible DO Regenerate scRNA Sequencing analysis

A. Left: Uniform manifold approximation and projection (UMAP) plot showing scRNA-seq data from (top) Inn DO (red) and Den DO (green) FACS-isolated hSSCs three months post distraction (Right) Four unique clusters of mSSCs are identified. Colors as labeled in the figure panel.
B. Feature plots depicting transcriptional expression of key genes implicated in mechanotransduction by hSSCs (top to bottom, left then right): PTK2, TAZ, YAP1, FOS, JUN, ATF4, GREM1, SOX9, PTH1R, DLX5
C. Feature plots depicting transcriptional expression of key genes implicated in Collagen 1 formation by hSSCs (top to bottom): COL1A1, COL1A2.
D. Feature plots depicting transcriptional expression of key genes implicated in Osteogenesis by hSSCs (top to bottom): RUNX2, SPP1, IBSP, BGLAP, IGF1.
E. Feature plots depicting transcriptional expression of key genes implicated in Neural Crest Proliferation and Differentiation by hSSCs (top to bottom): PAX3, MSX1, TWIST1, SOX9, SNAI2, RHOB.
F. Feature plots depicting transcriptional expression of key genes implicated in BMP signaling by hSSCs (top to bottom): ACTR1A, ACTR2, BMP4, SMAD4, SMAD7, BMPR1A.

Supplementary Figure 6. Human Mandible Distraction Regenerate scRNA Cluster Pathway and Gene Ontology analyses

A. Top left: Uniform manifold approximation and projection (UMAP) plot showing scRNA-seq data from (top) Inn DO (red) and Den DO (green) FACS-isolated hSSCs three months post distraction (Right) Four unique clusters of mSSCs are identified. Colors as labeled in the figure panel. (Below) EnrichR gene set enrichment analyses with graphs demonstrating pathways (determined by Bioplanet) associated with top 100 feature genes from each cluster 0, 1, 2, 3 as per p value ranking.
B. Top left: Uniform manifold approximation and projection (UMAP) plot showing scRNA-seq data from (top) Inn DO (red) and Den DO (green) FACS-isolated hSSCs three months post distraction (Right) Four unique clusters of mSSCs are identified. Colors as labeled in the figure panel. (Below) EnrichR gene set enrichment analyses with graphs demonstrating gene ontologies (determined by GO BP) associated with top 100 feature genes from each cluster 0, 1, 2, 3 as per p value ranking.

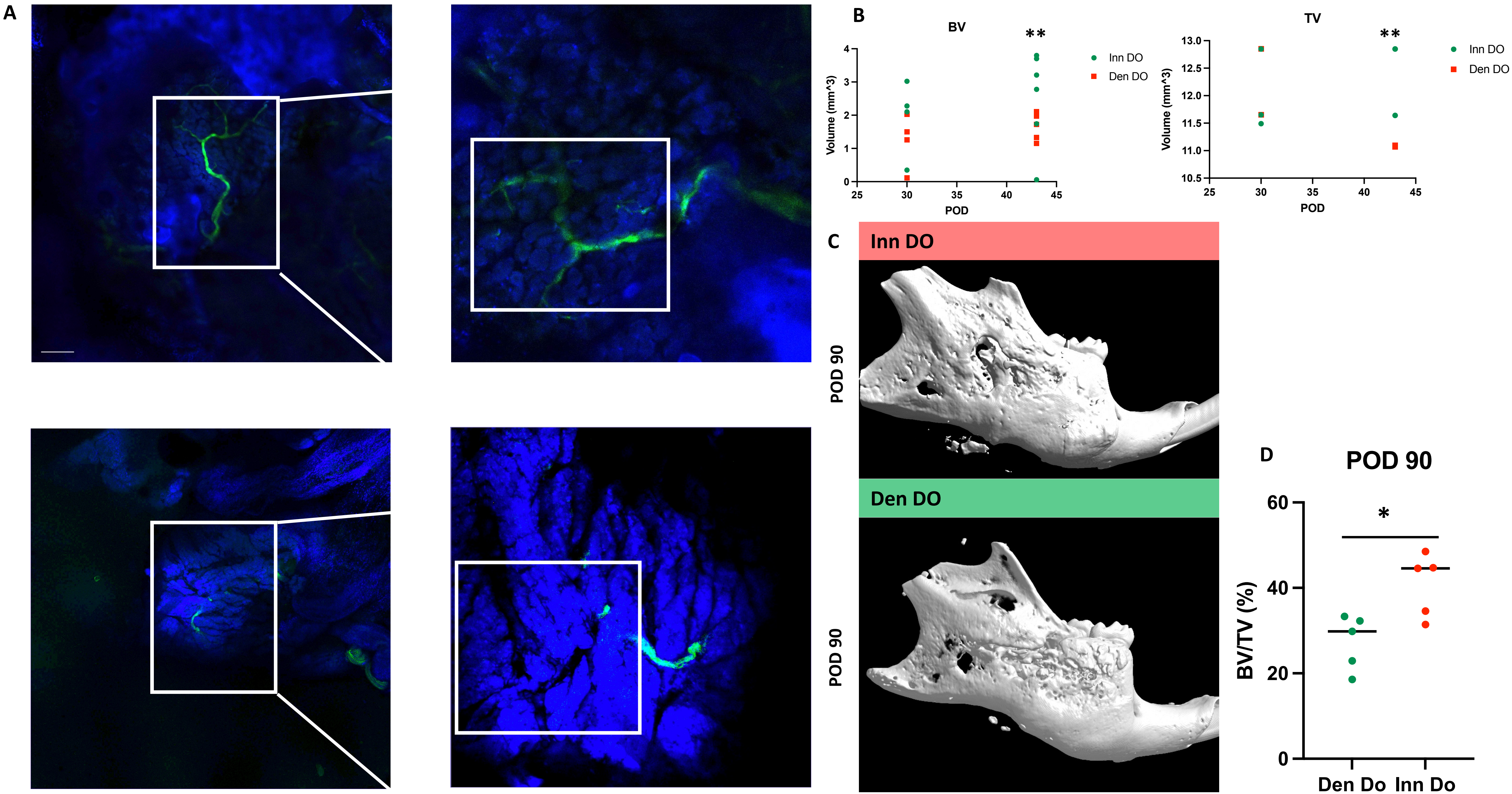

Supplementary Figure 1. Validation of Denervation injury model and POD 90 micro-CT reconstructions and analyses.

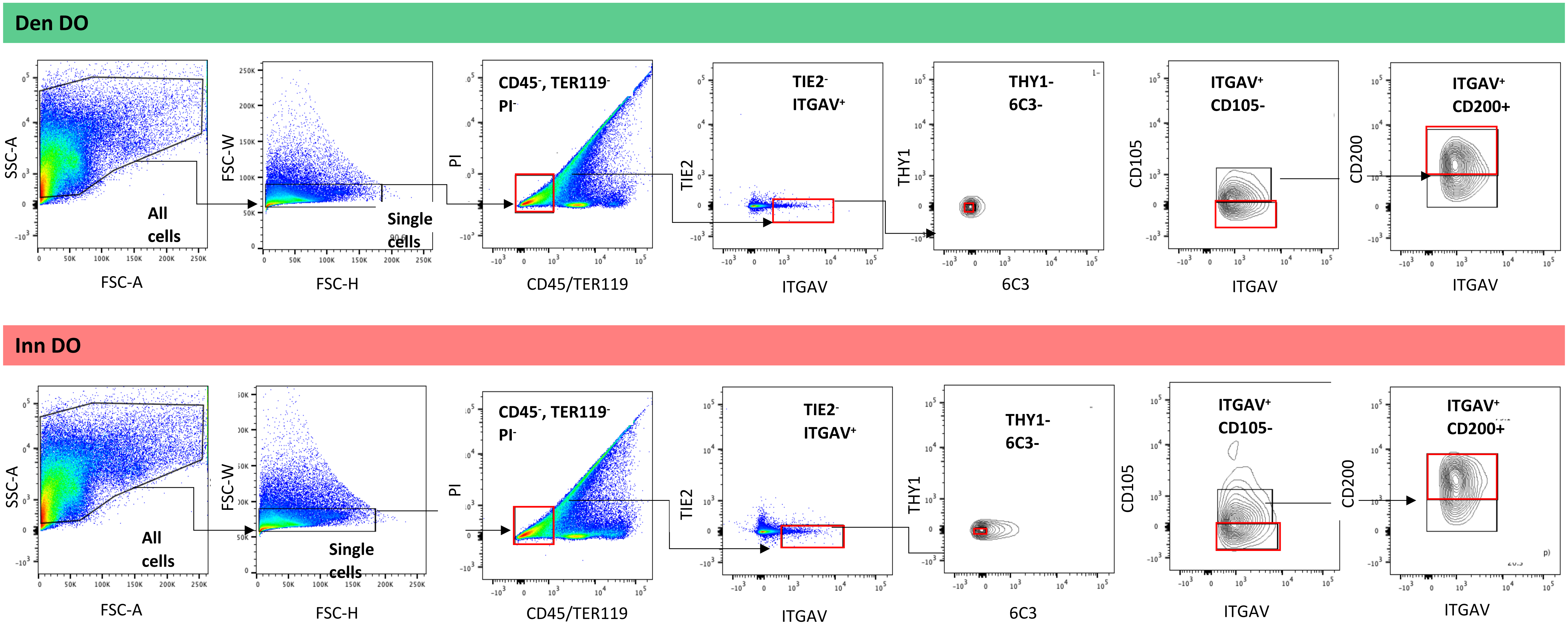

Supplementary Figure 2. FACS gating strategy for mSSC in DO Den and DO Inn Mandibles.

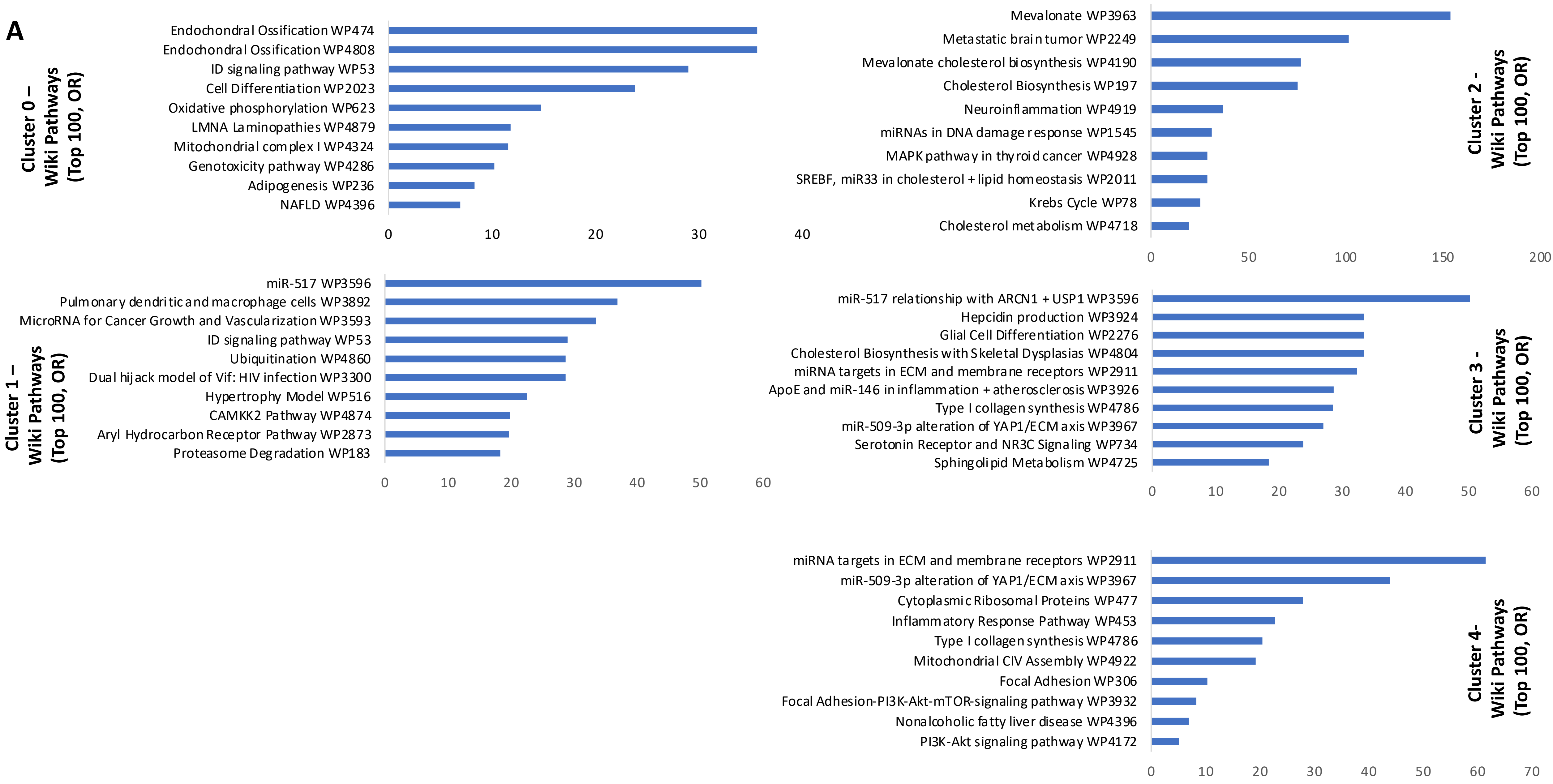

Supplementary Figure 3. Mouse Mandible Distraction Regenerate scRNA Cluster Pathway and Gene Ontology analyses

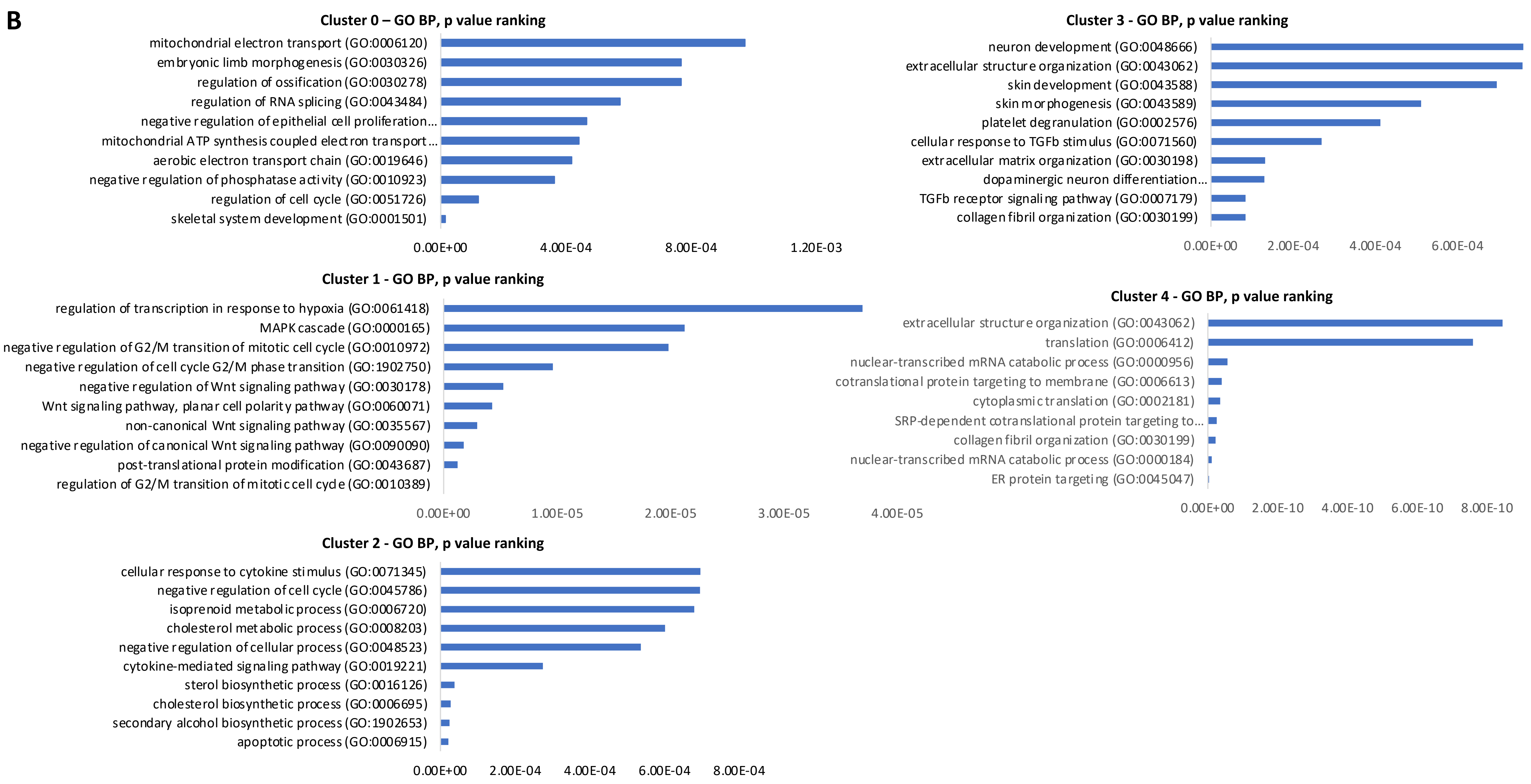

Supplementary Figure 3 cont. Mouse Mandible Distraction Regenerate scRNA Cluster Pathway and Gene Ontology analyses

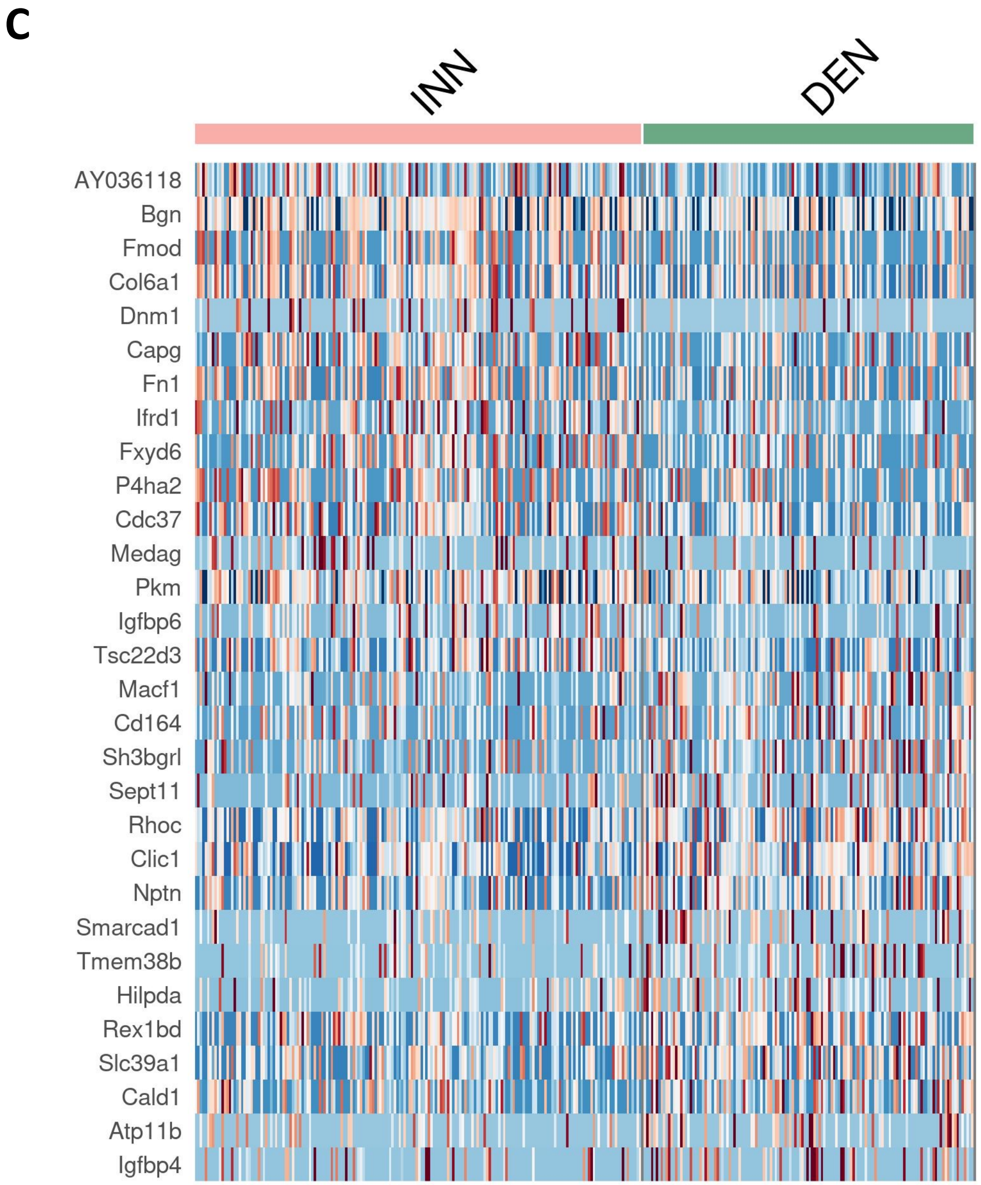

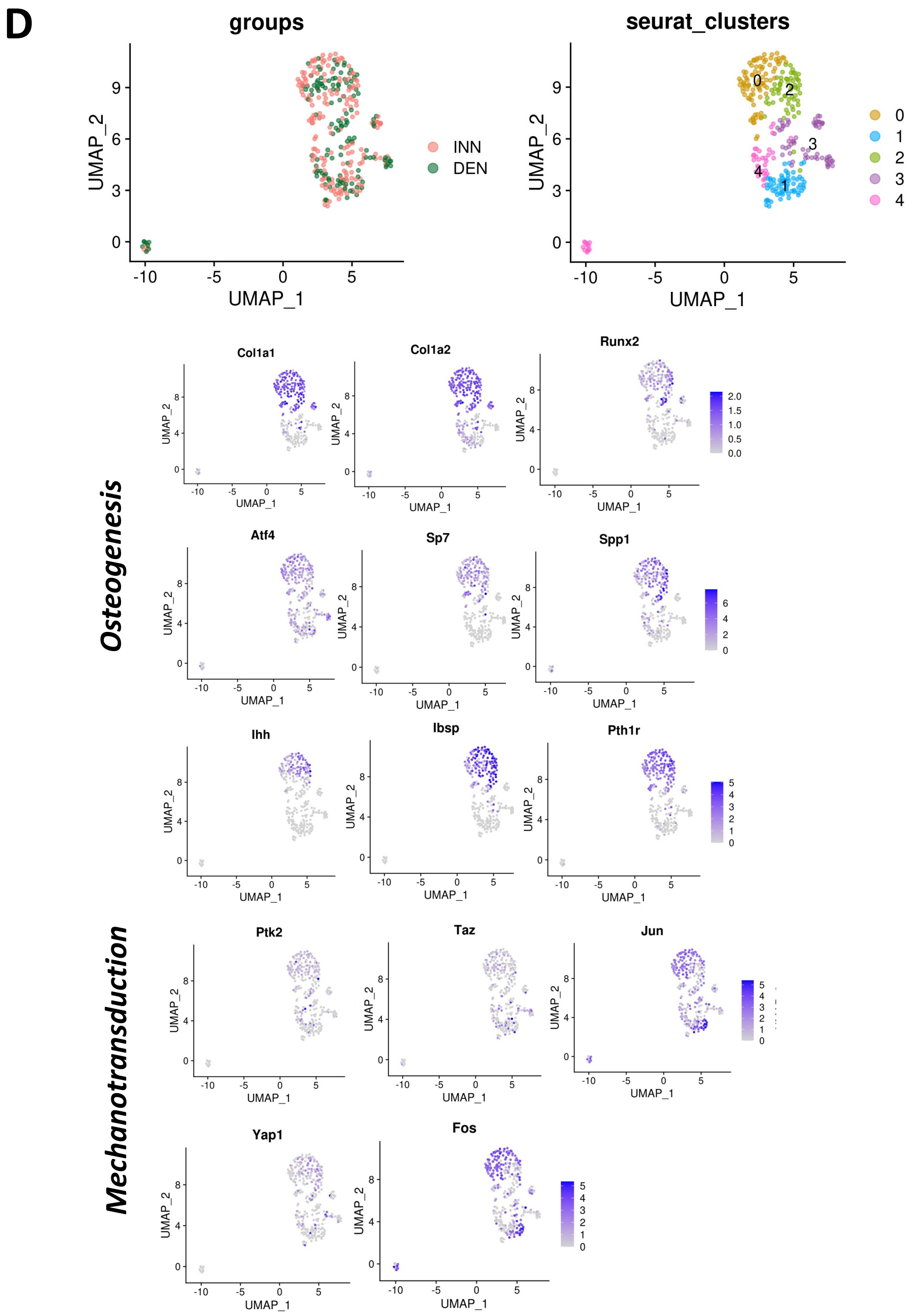

Supplementary Figure 3. Mouse Mandible Distraction Regenerate scRNA Cluster Pathway and Gene Ontology analyses

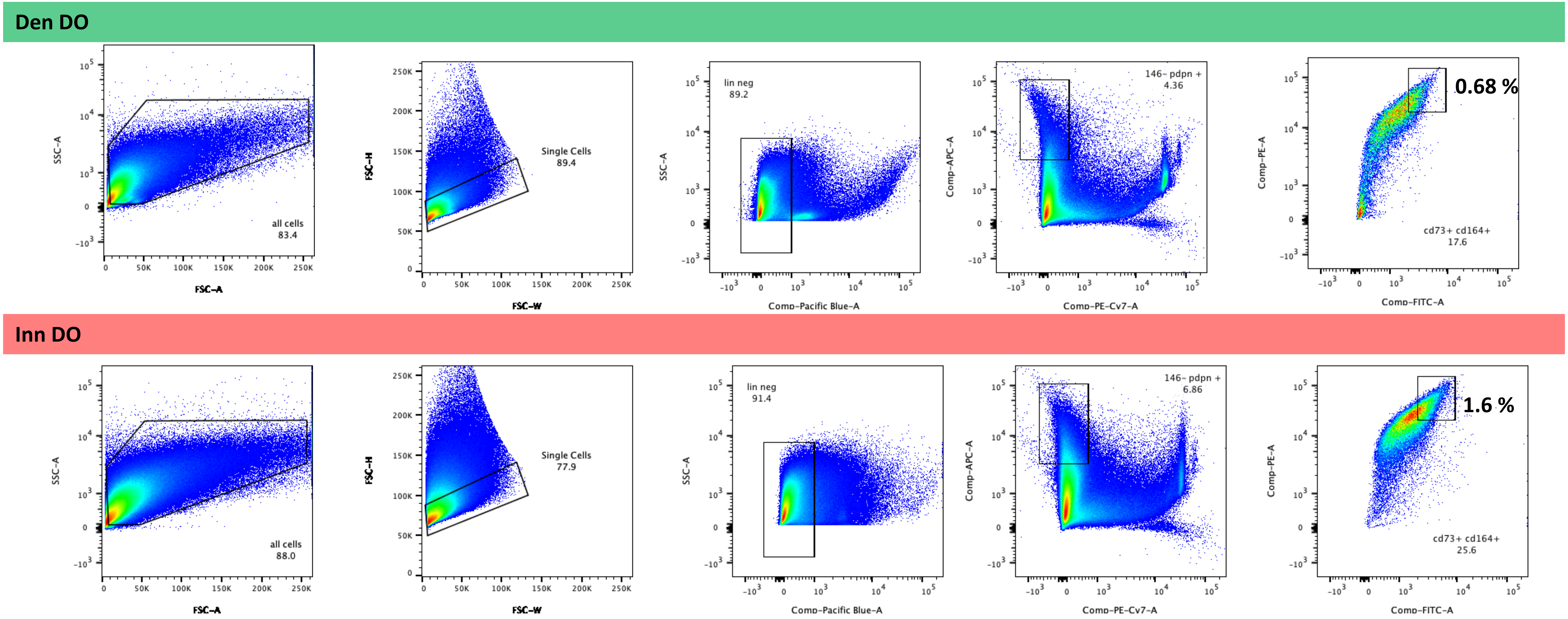

Supplementary Figure 4. Human Mandible Skeletal Stem Cell FACS Gating Strategy in DO Den (bottom) and DO Inn (top) Human Mandibles.

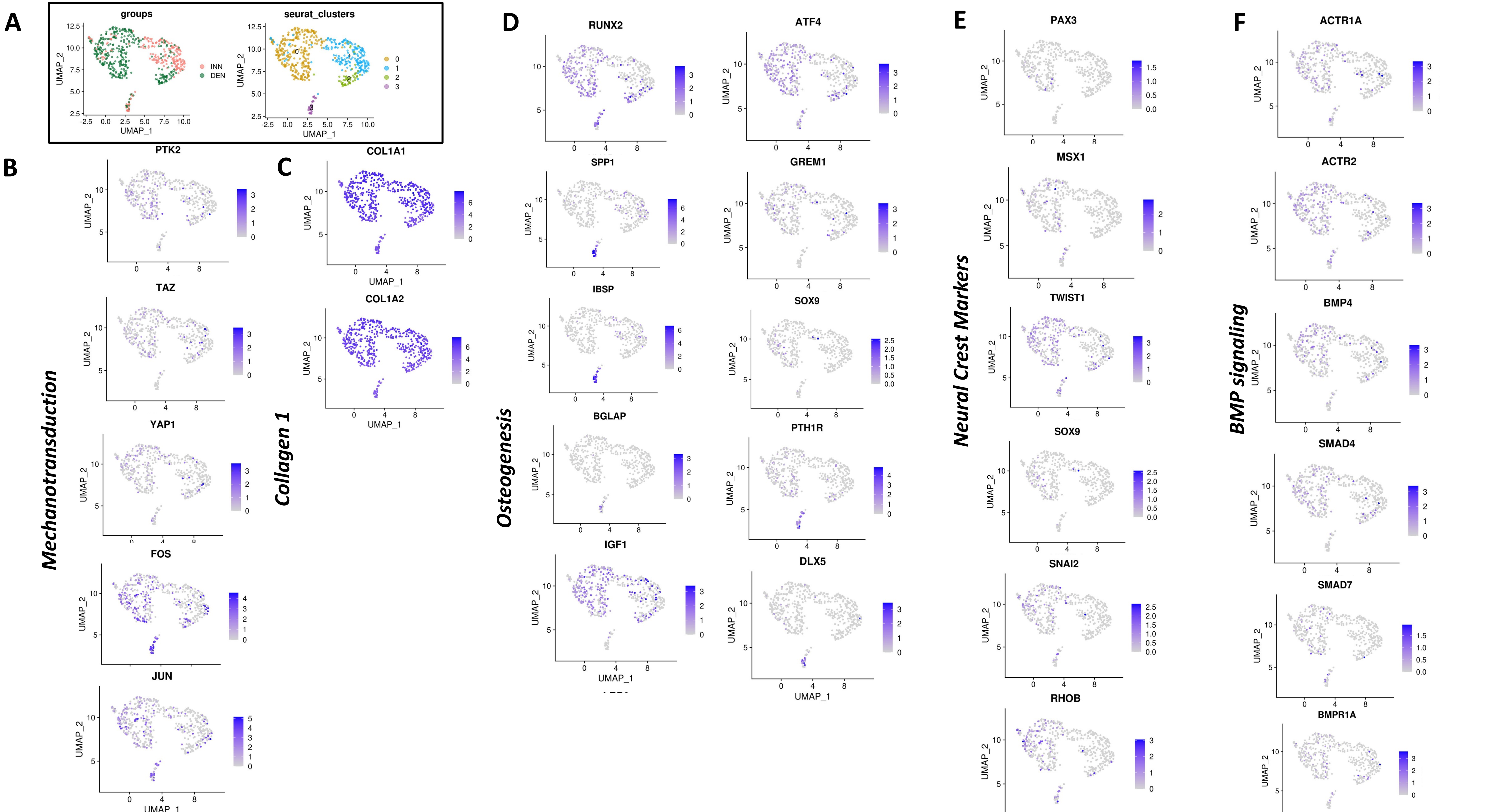

Supplementary Figure 5. Human Mandible DO Regenerate scRNA Sequencing analysis

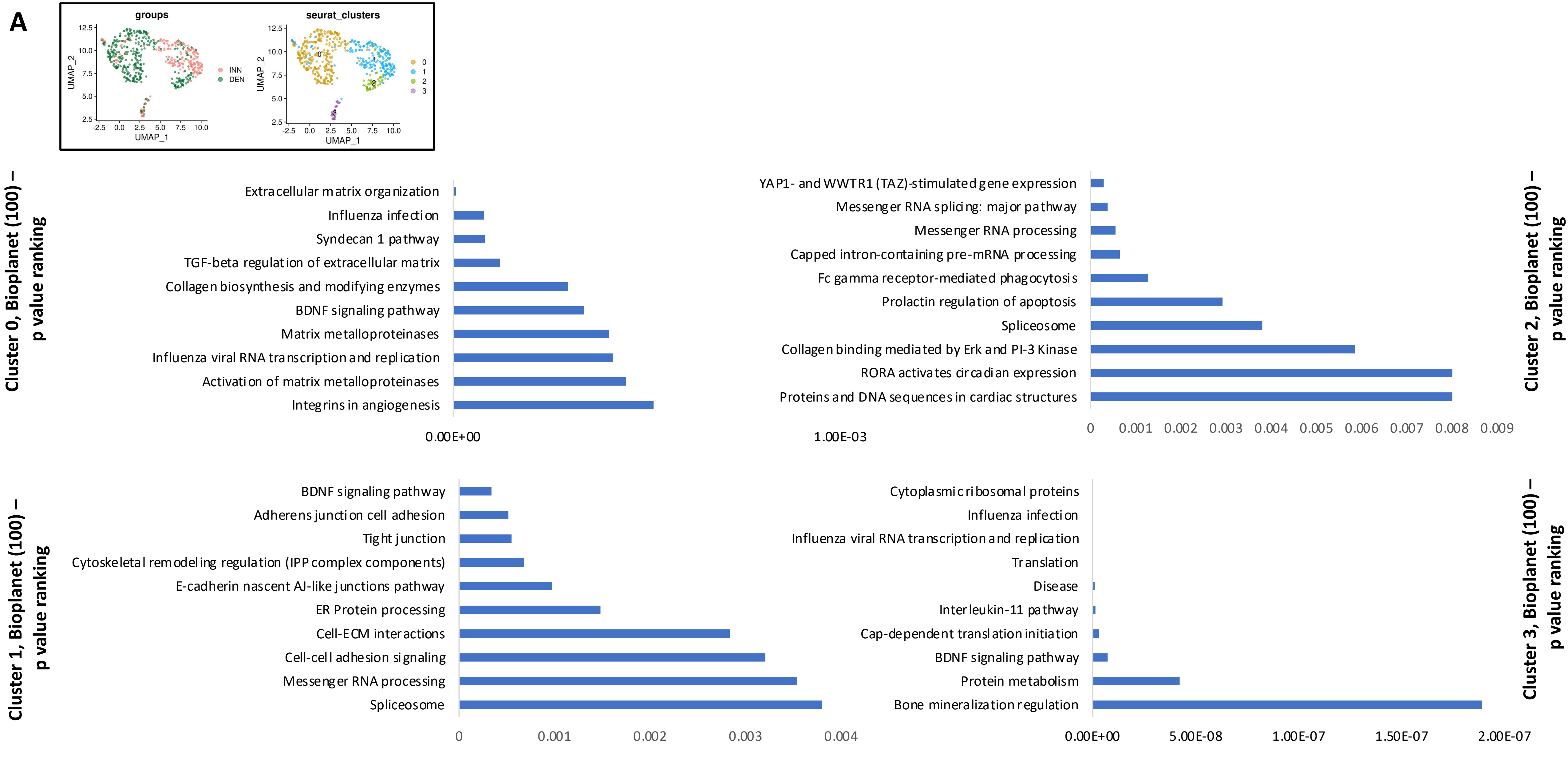

Supplementary Figure 6. Human Mandible Distraction Regenerate scRNA Cluster Pathway and Gene Ontology analyses

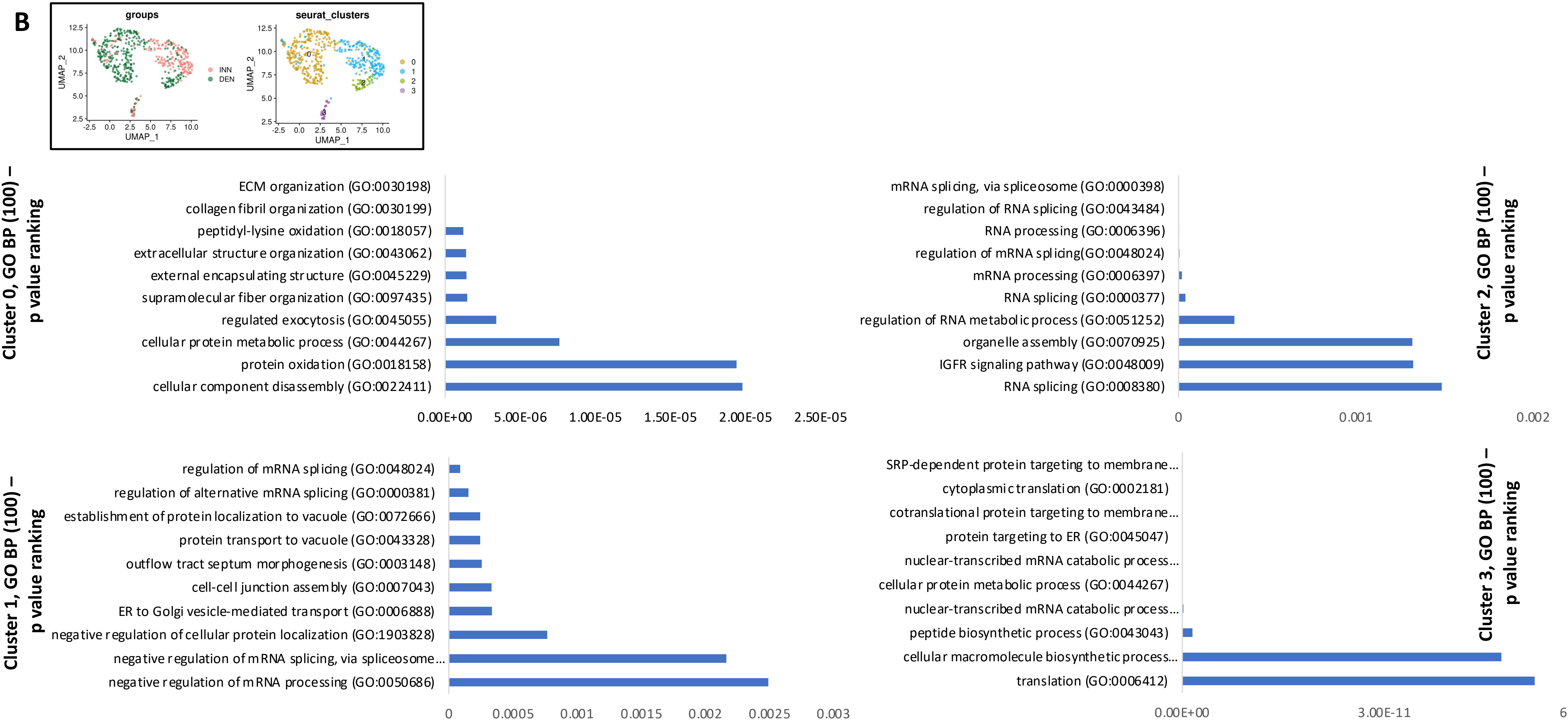

Supplementary Figure 6 cont. Human Mandible Distraction Regenerate scRNA Cluster Pathway and Gene Ontology analyses
